# A Systematic Review of Candidate Genes for Major Depression

**DOI:** 10.3390/medicina58020285

**Published:** 2022-02-14

**Authors:** Audrone Norkeviciene, Romena Gocentiene, Agne Sestokaite, Rasa Sabaliauskaite, Daiva Dabkeviciene, Sonata Jarmalaite, Giedre Bulotiene

**Affiliations:** 1 Clinic of Psychiatry, Institute of Clinical Medicine, Faculty of Medicine, Vilnius University, M. K. Ciurlionio Str. 21/27, LT-03101 Vilnius, Lithuania; matuseviciute.audrone@gmail.com (A.N.); romena.gocentiene@gmail.com (R.G.); 2 National Cancer Institute, Santariskiu Str. 1, LT-08660 Vilnius, Lithuania; agnesestokaites@gmail.com (A.S.); rasa.sabaliauskaite@nvi.lt (R.S.); daiva.dabkeviciene@nvi.lt (D.D.); sonata.jarmalaite@nvi.lt (S.J.)

**Keywords:** major depression, candidate genes, genetic associations, gene polymorphism

## Abstract

*Background and Objectives:* The aim of this systematic review was to analyse which candidate genes were examined in genetic association studies and their association with major depressive disorder (MDD). *Materials and Methods:* We searched PUBMED for relevant studies published between 1 July 2012 and 31 March 2019, using combinations of keywords: “major depressive disorder” OR “major depression” AND “gene candidate”, “major depressive disorder” OR “major depression” AND “polymorphism”. Synthesis focused on assessing the likelihood of bias and investigating factors that may explain differences between the results of studies. For selected gene list after literature overview, functional enrichment analysis and gene ontology term enrichment analysis were conducted. *Results:* 141 studies were included in the qualitative review of gene association studies focusing on MDD. 86 studies declared significant results (*p* < 0.05) for 172 SNPs in 85 genes. The 13 SNPs associations were confirmed by at least two studies. The 18 genetic polymorphism associations were confirmed in both the previous and this systematic analysis by at least one study. The majority of the studies (68.79 %) did not use or describe power analysis, which may have had an impact over the significance of their results. Almost a third of studies (N = 54) were conducted in Chinese Han population. *Conclusion:* Unfortunately, there is still insufficient data on the links between genes and depression. Despite the reported genetic associations, most studies were lacking in statistical power analysis, research samples were small, and most gene polymorphisms have been confirmed in only one study. Further genetic research with larger research samples is needed to discern whether the relationship is random or causal. *Summations:* This systematic review had summarized all reported genetic associations and has highlighted the genetic associations that have been replicated. *Limitations:* Unfortunately, most gene polymorphisms have been confirmed only once, so further studies are warranted for replicating these genetic associations. In addition, most studies included a small number of MDD cases that could be indicative for false positive. Considering that polymorphism loci and associations with MDD is also vastly dependent on interpersonal variation, extensive studies of gene interaction pathways could provide more answers to the complexity of MDD.

## 1. Introduction

Major depressive disorder (MDD) is a common psychiatric illness accompanied by high levels of morbidity and mortality. MDD causes major psychological, physical, and social impairments [1]. It can cause the affected person to suffer greatly and function poorly at work, at school and in the family. According to the World Health Organization (WHO), at a global level, more than 264 million people are estimated to suffer from depression [2]. Depression is ranked by WHO as the single largest contributor to global disability (7.5% of all years lived with disability (YLDs) in 2015). Along with population growth and aging, many cases of depression overloaded healthcare systems, thereby generating the need for resource optimization [3]. There is a clear need to identify prognostic indicators that could be used to select individuals at higher risk of developing MDD in order to aid the management of patients in clinical practice. Identifying risk variants using genetic analysis and thereby increasing our understanding of how MDD arises, could lead to improved prevention and the development of new and more effective therapies [4].

Even though information concerning the epidemiology and symptoms of depression are well documented, the current understanding of the aetiology and pathophysiology of MDD are still rudimentary [5]. It is known that depression results from a complex interaction of social, psychological, and biological factors [6]. Genetic factors substantially contribute to MDD, as indicated by family, twin, and adoption studies [7]. For instance, a meta-analysis of twin research data shows that the heritability rate for depression is 37% and data from family studies show a two- to threefold increase in the risk of depression in first-degree offspring of patients with depression [8]. MDD is a genetically complex disease. Only a small number of genes have been proven to be associated with MDD development risk [9].

Available literature regarding the genetics of MDD is vast and complex. Researchers have taken upon themselves the task of determining the genetic architecture of MDD using different molecular approaches, including the linkage and genome-wide association (GWA) studies. Recent GWA meta-analysis of 135,458 MDD cases and 344,901 controls, identified 44 independent and significant risk loci for MDD [10]. Recent study [11] which identified MDD candidate genes and performed association studies at the polymorphism and gene level in multiple large samples, found no support for candidate gene or candidate gene-by-interaction hypotheses for major depression. A comprehensive systematic review of linkage studies was done in 2012, in which the authors stated that the results of explored studies lacked significant findings in any candidate gene meta-analysis [12].

Our aim was to analyse which candidate genes have been studied in the genetic association studies and identify their association with major depressive disorder (MDD). In order to update our knowledge of recent findings of linkage studies, this systematic review will explore available data regarding candidate genes for MDD from July 2012 until March 2019.

## 2. Materials and Methods

### 2.1. Literature Selection

The protocol for this systematic review has been registered in the international prospective register of systematic reviews (PROSPERO protocol ID: CRD42019129194 available at https://www.crd.york.ac.uk/PROSPERO/display_record.php?RecordID=129194 (accessed on 9 December 2022). We conducted a systematic literature search to update genetic case-control linkage studies on MDD, using a gene-candidate approach with those published between 1 July 2012 and 31 March 2019; the end search date was 23 March 2019 [13] (e PUB publication) using MEDLINE1 via PUBMED. The search strategy we first developed in MEDLINE (OVID) and then adapted in PubMed. Two investigators (AN and RG) independently conducted a literature search in PubMed for relevant articles between September 2019 and February 2020. The search strategy included articles published from July 2012 until March 2019 using the following combinations of relevant keywords: “major depressive disorder” OR “major depression” AND “gene candidate”, “major depressive disorder” OR “major depression” AND “polymorphism”. Titles, abstracts, and full texts we screened sequentially for eligibility criteria and any discrepancies were resolved by consensus or by a third reviewer (GB). Data we crosschecked to ensure consistency. Inclusion criteria were as follows: (i) the patients had a primary diagnosis of major depressive disorder; (ii) the study examined the association between a candidate gene (a SNP) and MDD; (iii) the study was a case-control study; and (iv) the study was published in the English language. Exclusion criteria: (i) non-human studies; (ii) non-genetic studies; (iii) other than case-control study design; (iv) another genetic approach than gene candidate association analysis used; (v) enrolled patients did not have a primary diagnosis of major depressive disorder or had psychiatric comorbidities; (vi) studies focusing on treatment of MDD; (vii) articles with insufficient data for analysis reported (only abstract available). We excluded the abstracts that did not mention investigating the genetic association(s) between MDD and one or more genetic polymorphisms.

### 2.2. Data Synthesis

A descriptive and tabular synthesis was carried out using the extracted data and major findings of each included study. A team of three members extracted the data. The data included: (1) author information, (2) year of publishing, (3) information on the setting for each study (the genotyping method employed, statistical model, and statistical detected power), (4) characteristics of study participants (phenotypic definitions and geographic characteristics), (5) characteristics of candidate gene (type, locus, and evidence of functional role), (6) outcome measure (raw *p*-values and odds ratios (ORs) for genotypes and/or allele frequencies and the corrected results if the study had applied corrections for multiple testing, Hardy–Weinberg equilibrium (HWE) test). A “Tool to Assess Risk of Bias in Case Control Studies” [14], which is recommended by CLARITY group, was used to assess the quality of all eligible studies. Based on the tool, each study was assessed in five dimensions (assessment of exposure, outcome development, case and control subject selection, group comparability) and appointed to one of the three categories: of low, higher, and high bias, which stratified the studies into studies of low, higher, or high risk of bias. Examples of low risk of bias: comprehensive matching or adjustment for all plausible prognostic variables; from a data base with documentation of accuracy of abstraction of prognostic data. Examples of higher risk of bias: matching or adjustment for most plausible prognostic variables; data base with uncertain quality of abstraction of prognostic information. Examples of high risk of bias: Prognostic information from data base with no available documentation of quality of abstraction of prognostic variables; matching or adjustment for a minority of plausible prognostic variables, or no matching or adjustment at all. Statements of no differences between groups or that differences were not statistically significant are not sufficient for establishing comparability.

If sufficient information could not be extracted and bias could not be assessed, those studies were considered as having an unclear risk of bias. Synthesis focused on describing the direction and consistency of effect, assessing the likelihood of bias, and investigating factors that may explain differences between the results of studies.

### 2.3. Gene Functional Enrichment Analysis

For the selected gene list after the literature overview, a functional enrichment analysis was performed using WEB-based Gene Functional Classification tool (DAVID Bioinformatics Recourses 6.8). Gene Ontology (GO) term enrichment analysis was conducted using medium classification stringency, and significance value was adjusted by the false discovery rate (FDR) analysis using the Benjamini–Hochberg (BH) procedure. GO terms were assigned to one of three categories, Biological process (BP), Molecular function (MF), and Cellular component (CC) terms, and only included in further analysis if statistically significant (*p* < 0.05).

## 3. Results

### 3.1. Literature Search

**Figure 1:**
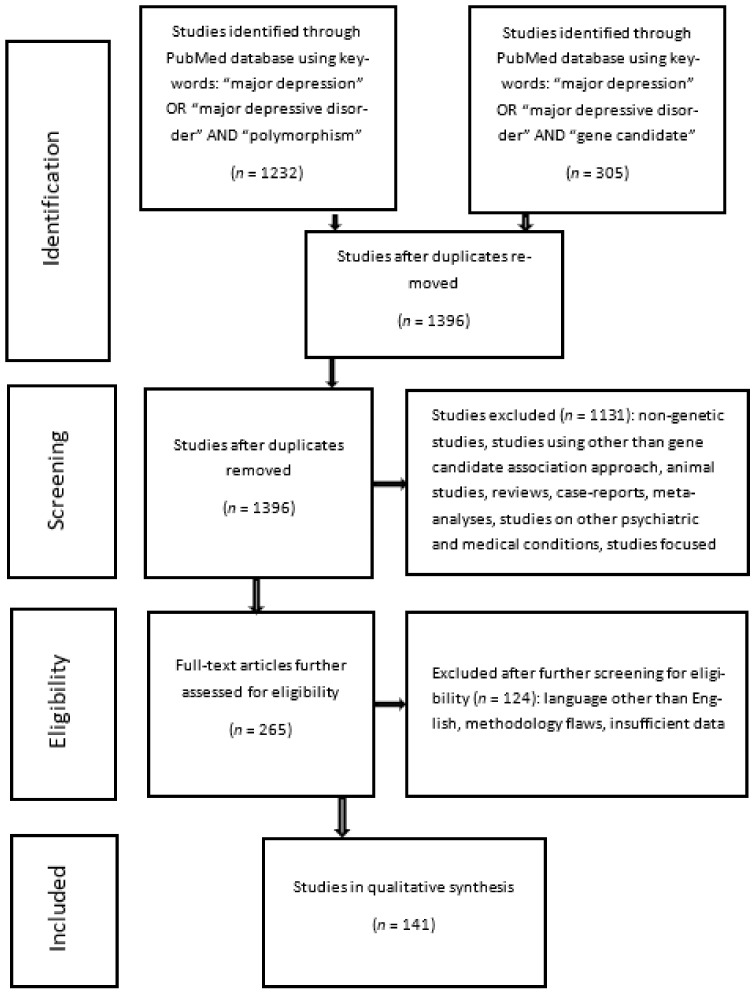
Flowchart of literature review process.

The first search strategy on PubMed using the following keywords: “major depression” OR “major depressive disorder” AND “polymorphism”, identified a total of 1232 studies, and the second search strategy using the following keywords: “major depression” OR “major depressive disorder” AND “gene candidate”, identified 305 studies, of which 141 were duplicates. After the removal of duplicates and 1255 hits not fulfilling the inclusion criteria, 141 studies were finally included in the qualitative review of gene association studies focusing on MDD. The detailed flowchart of the literature review process we reported in Figure 1.

### 3.2. Summary of Eligible Studies

We present a narrative summary on investigated candidate genes for MDD reported from July 2012 until March 2019. We have summarized the main characteristics of the identified studies in Table S1 (Table S1: Main characteristics and findings of studies included in the systematic review.)

Only significant *p* values (abbr. *p*) and odds ratios (abbr. OR) are presented in Table S1. Selected articles explored associations (see Table S1) of 595 polymorphisms in 175 genes. The 87 articles reported nominal significant associations (*p* < 0.05). These 87 articles reported significant results for 172 polymorphisms in 85 candidate genes. In 25 of these genes, multiple SNPs associations were found by one or more studies. The 13 SNPs associations were confirmed by at least two studies. Other SNPs associations were confirmed only by one study, 20 of those delivered conflicting results. Two studies [15,16] delivered results of meta-analyses, in which two SNPs showed significance. Four studies found significant associations only after population stratification by gender: in the women subgroup [17,18], in the men subgroup [19,20]; one study enrolled only female participants [21]; and one study presented significant results in a particular age group of 37 years or older participants [22]. The genotyping methods and main tests for statistical analyses used in the studies are summarized in Table S2 (Table S2: Summary of genotyping and statistical methods used in the eligible studies).

We observed that 31% of all studies reported using quantitative polymerase chain reaction (qPCR); other methods were used in 15% or less of the eligible studies. The 80 out of 141 studies (57%) applied a method to correct for testing multiple gene variants. Association remained significant after correction for multiple testing in 35 studies. Most of the articles (122 out of 141) reported examining HWE for studied polymorphisms. According to the authors, the results showed that in most studied populations both case and control groups were in HWE, in some of these studies SNPs not in accordance with HWE were exempted from further analysis [23,24,25,26,27,28,29,30,31,32,33,34,35]. Fifty-four of the eligible studies were performed in China, 13 in Poland, 11 in Korea, 5 in Japan, 4 in the USA, Spain, Germany, Italy, Denmark, and Turkey, 3 in the Netherlands, 2 in Australia, Malaysia, Taiwan, Iran, Slovakia, Hungary, and the United Kingdom, and 1 in the other 17 countries of Europe, Asia, or South America.

Ten (7%) studies were evaluated to be in the high risk of bias group; 35 (25%) had a higher risk of bias; 61 (43%) had a low risk of bias; and 35 (25%) studies could not be sufficiently evaluated and were stated as having unclear risk of bias (for detailed assessment information see Supplementary information, Section A).

It is worth mentioning that 97 out of 141 studies (69%) did not use or describe power analysis, which may have had an impact over the significance of their results, as some of the authors discuss in their articles.

### 3.3. Comparison of Results with the Previous Systematic Analysis Regarding Associations between Gene Candidates and MDD

The previous comprehensive systematic review of linkage studies regarding MDD was published in 2012 [12]. After comparing findings of previously analysed studies with selected articles of our review, only 18 genetic polymorphism associations were confirmed in both reviews by at least one study. Most of the recent studies analysed other SNP’s. Both reviews included studies with various sample sizes; however, it is important to note that the majority were lacking in power analysis. The previous review also conducted a replication study with a considerably large sample size. They found that 13 SNPs in 12 genes showed significant associations with MDD in the full sample. Twp of those SNP’s (rs1360780 and rs2522833) were further tested and significant associations in two studies from our review were confirmed.

### 3.4. Results of Gene Functional Enrichment Analysis

**Figure 2:**
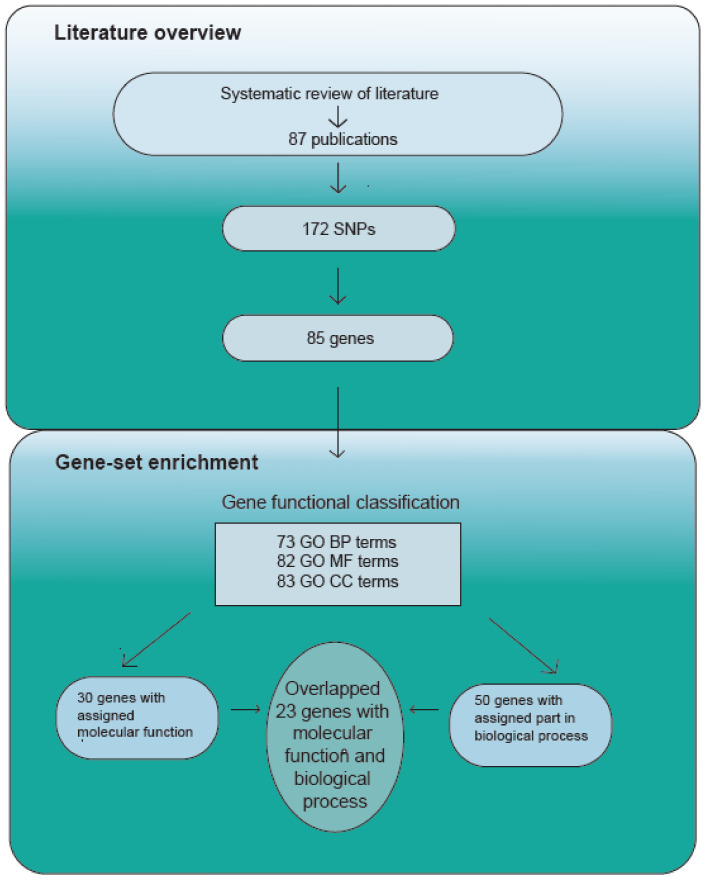
Experiment scheme.

To explore the biological knowledge of genes associated with MDD (*n* = 85) in BP, MF and CC and molecular pathways, we used GO and KEGG enrichment. The functional annotation of GO was successful with assigned 73 GO Biological process, 82 GO Molecular function and 83 GO Cellular component terms (Figure 2). Additionally, KEGG pathway enrichment was performed, and we determined that 16 of these MDD associated genes are primarily involved in neuroactive ligand–receptor interaction (KO04080)—which may be an indication of highly important differentiated brain activity during MDD (for detailed outline of investigated genes (see Supplementary information, Section B).

Further analysis was conducted only with genes with assigned significant (*p* < 0.05) molecular function and biological process (*n* = 30 and *n* = 50).

Findings from DAVID-GO term and KEGG pathway analysis were further refined.

**Figure 3:**
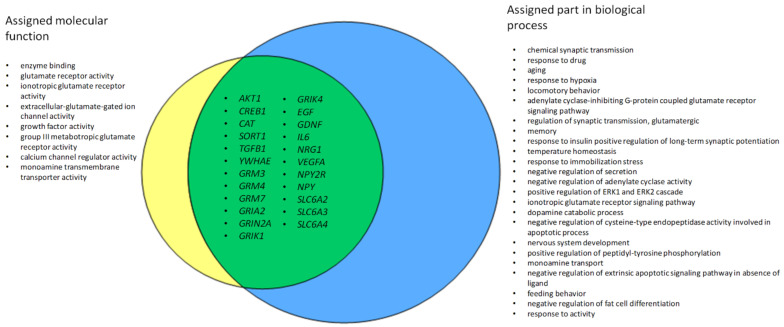
Overlapped genes from molecular function and biological process enrichment analysis associated with MDD.

We overlapped significant genes from both MF and BP groups and identified 23 genes that could be related to MDD based on our sample of studies (Figure 3).

The analysis of significant MF from GO showed that MDD-associated genes mainly function as enzyme binding (GO:0019899) (*n* = 11, e.g., *AKT*, *CREB1*, *CAT*, *SORT1*, *TGFB1*, *YWHAE*), as receptors (e.g., glutamate receptor (GRM) family or receptor subunits (GO:0008066, GO:0004970, GO:0001642) (*GRIA*, *GRIN*, *GRIK*) or are involved in transportation (GO:0022857) such as *SLC6A* family. The majority of ascertained significant BP were associated with the chemical synaptic transmission (*n* = 17, GO:0007268). Furthermore, genes significantly related with CC were with the key word “membrane” in the attributed terms (GO:0005887, GO:0042734, GO:0045211, GO:0016021) (more information in Supplementary information, Section B).

## 4. Discussion

### 4.1. Characteristics of Most Studied Genes

In the present study, we analysed 141 publications that performed candidate gene association studies to determine MDD associated SNPs. Intensive literature analysis, we also performed on most prominent genes to further unravel possible gene candidate association with MDD. We examined significant 172 polymorphisms in 85 candidate genes. We found the top 23 genes with successfully assigned molecular function and biological process that in GO terms could be involved in brain disorders such as major depressive disorder. The in-depth analysis revealed that the most crucial genes for MDD could be *GRIA*, *GRIN*, and *GRIK* family genes as well as more well-known *SLC6A* family members. However, it is important to stress that associations with all these candidate genes were found mostly in single studies, so replication studies are crucial in order to determine the significance of these results.

#### 4.1.1. Genes Involved in the Glutamatergic Pathway

Gene functional analysis from MDD associated genes revealed that the majority of genes from this sample of studies are involved in signal transmission, especially glutamate neurotransmission. Glutamate receptors genes such as *GRIA2* (glutamate ionotropic receptor AMPA Type Subunit 2), *GRIN2A* (glutamate ionotropic receptor NMDA type sub-unit 2A), *GRIK1* and *GRIK4* (glutamate ionotropic receptor kainate type sub-unit 1 and 4) are ligand activated ion channels which allow ions to flow to the neurons upon activation. The metabotropic glutamate receptor (mGluR) genes, namely, *GRM3*, *GRM4,* and *GRM7* (glutamate metabotropic receptor 3, 4, and 7) are G-protein-coupled receptors which enable cell activation by extracellular signalling molecules [36]. Several authors from our review investigated and found associations between gene candidates involved in the glutamatergic pathway; however, the lack of power analysis in some studies with potentially too small sample sizes does not allow us to view the results as more promising [15,37,38,39].

#### 4.1.2. Genes Involved in Neurotransmition through Regulation of Calcium Channel Activity

Other genes researched in the studies of our systemic review, encode proteins involved in neurotransmission through regulation of calcium channel activity, such as *NPY* (neuropeptide Y) and *NPY2R* (neuropeptide Y receptor Y2), while others—*SLC6A2*, *SLC6A3,* and *SLC6A4* (solute carrier family 6 member 2, 3, and 4) are responsible for monoamine transmembrane transporter activity. According to recent literature, NPY was associated with the resistance to treatment in MDD [40]. One study in our review investigated and found a positive association for a few polymorphisms of NPY (33); it also had a quite large sample of participants; however, power analysis was not performed or described. NPY receptor gene *NPY2R* previously had been reported to be involved in neurodegenerative disorders such as Huntington’s disease [41]. Only 1 study in our review investigated this gene [42]. Despite a determined statistically positive association, it was likely lacking in statistical power due to a small sample size.

Among calcium channel regulators, *SLC6A4* is one of the most widely studied genes. It is responsible for transportation of serotonin. The repeat polymorphism with long (L) and short (S) alleles of this gene could be associated with depression and response to treatment [43,44]. In a recent report, family members 2 and 3 of this gene family have been also identified as MDD candidate genes [45]. Studies included in our review further investigated associations between MDD and polymorphisms of genes like *SLC6A2, SLC6A3,* and *SLC6A4*. However, the results of those studies were conflicting. For example, some studies with various samples showed positive association for polymorphisms of SLC6A4 [22,46,47,48,49,50,51,52,53] while others did not confirm such findings [54,55,56,57,58]. The same tendency was seen for the polymorphisms of SLC6A2 [59,60,61], which may indicate a possibility of false positive results. As for SLC6A3, 1 study found a significant association of 1 polymorphism in a moderate sample of participants [59].

#### 4.1.3. Genes Involved in Apoptosis

Some genes with assigned molecular function of enzyme binding or growth factor activity were also attributed to the biological process of apoptosis. Specifically, AKT1 (AKT serine/threonine kinase 1), SORT1 (sortilin 1), YWHAE (tyrosine 3-monooxygenase/tryptophan 5-monooxygenase activation protein epsilon), GDNF (glial cell derived neurotrophic factor), NRG1 (neuregulin 1), and VEGFA (vascular endothelial growth factor A). Apoptosis could be considered as one of the main metabolic pathways related to MDD pathophysiology [62]. Apoptosis suppressors, including AKT1, GDNF, VEGFA, and NRG1, have been previously associated with MDD. AKT1 gene polymorphisms were associated with MDD severity [63]. Another apoptosis blocker is GDNF that promotes the differentiation, maintenance, and viability of various cell populations and thus neuronal survival in the nervous system [64]. It is especially important to mention GDNF interaction with the brain neurotransmitters—specifically, previously mentioned AMPA receptors, and this cross-talk could be important in the pathogenesis of depression [65]. The role of VEGFA in the nervous system is very similar to GDNF. The growth factor in certain conditions is involved in neuronal survival by increasing the proliferation and decreasing the apoptosis of neural progenitors, and the polymorphic variation in VEGFA might explain the variability in response to treatment [66]. Meanwhile, NRG1—a family of extracellular growth factors are responsible for maturation and migration of neurons that simultaneously block cell death through interactions with their tyrosine kinase receptor on the neural cell surface [67]. In a previous study, polymorphic variants of NRG1 was associated with predisposition for bipolar disorder [68]. On the other hand, apoptosis inducer sortilin encoded by the SORT1 gene located on chromosome 1p13.3 was also associated with other neurotrophic factors, such as VEGFA [69]. Finally, in other neural disorders–for instance, suicidal behaviour and schizophrenia, the apoptosis stimulator YWHAE, which encodes eta-polypeptide 14-3-3ε, haplotype (rs1532976), indicated more pronounced suicidal behaviour or risk of disease occurrence [70]. We also investigated in the reviewed studies, polymorphisms of the discussed genes. Studies for NRG1 delivered conflicting results, with a larger sample size in the study which found a positive association [27,71]. YWHAE was also investigated in a large study [19] with significant statistical power and found a positive association. Other studies which investigated VEGFA [72], GDNF [28], AKT [34], and SORT [69] were lacking in statistical power so it is likely that replication studies with larger samples will be needed in order to find out if these genes have any substantial impact on development of MDD.

There are many reasons why pinpointing the SNPs in MDD is very difficult. MDD is presumably a polygenic disease impacted by many genetic variations, as other well studied neural diseases such as schizophrenia [10]. Considering this, many of the genes associated with MDD may not be responsible for a causal effect of the disease as previously mentioned by Border et al. [11], but may be involved in interconnecting gene effect pathways and in treatment resistance. Interpersonal variation also plays an important role in manifold genetic associations identified in MDD. Considering this, extensive studies of gene interaction pathways could provide more answers to the complexity of MDD.

It is interesting that only a few polymorphisms were further studied after the 2012 systematic review, and even fewer gained any evidence in their favour. This may show trends of investigators mostly choosing new fields. It is also possible that the investigation of gene candidates as a method of choice may not be that promising in finding out the genetic biomarkers of MDD, as a rather recent large replication study of candidate genes suggests [11].

Most recent research 2019–2021 was focused on genes such as MTHFR [73] and BDNF [74]. The literature review (Fratelli at all, 2020) [75] showed the association between the 5HTTLPR genetic variants and several aspects of MDD. These findings show a consistently similar trend of genetic biomarker selection for further investigation as in our review.

### 4.2. Important Characteristic of Included Studies

We consider the major design weaknesses of the analysed studies as follows: lack of matching or adjustment for gender and age between case and control groups, small sample sizes and lack of statistical power analysis, as well as lack of description concerning used statistical analysis.

Because many studies were small, and most of all eligible studies have not reported sample size estimation or power analysis, they were prone to type-II error. Because the reliability of the results is affected by the sample size, the lack of statistical power analysis in most studies leaves some uncertainty about the significance of the results.

We observed a substantial heterogeneity among the included studies. Studies differed widely on their population characteristics, case definitions, selection of controls, and statistical and genotyping analyses. In most cases, samples were collected from only one hospital, which increases the selection bias. Some studies did not provide sufficient description of methods used in their statistical analysis. Considering this and striving for good transparency, we carefully considered the risk of bias, but by trying to lessen the effect of those limitations and increase the review comprehensiveness, we did not exclude high risk-of-bias studies. In assessing the risk of bias in these studies, we specified risks specific to this content area. As described previously, each study we assessed in five dimensions and the fifth dimension addressed the question if the statistical adjustment was carried out for two variables: gender and age. Some of the studies with significant differences between case and control groups included in our review did not make an adjustment based on their gender and age in their statistical analysis. Because gender and age are important in the onset of depression, the lack of correction may have influenced the significance of the results.

In addition, we should mention that in many studies results were not consistently corrected for multiple testing, so their findings were prone to type-I error.

We should consider that the results might have been influenced by the fact that deviation from Hardy–Weinberg equilibrium (HWE) was detected in some studies and was not even calculated in others. Deviation from HWE in MDD subjects could be a result of a sampling bias or genotyping error. Alternatively, this could indicate that those SNPs are causative variants for MDD. This latter possibility should be evaluated in further studies using additional independent populations.

We found that more than two thirds of candidate genes were investigated in the Han Chinese population, and less studied in individuals of Western European ancestry or Mexican-American populations. In comparison, the previous systematic review, which analysed the September 2007 and June 2012 studies [12], was dominated by a Western European population. This could influence the generalization of the results. Research showed that due to natural selection, genetic heterogeneity of susceptibility to complex diseases, such as affective disorders, intensifies. In addition, there are inconsistencies in detecting the genetic markers of these diseases among different ethnic populations.

### 4.3. Strengths and Limitations of This Review

The strengths of this study include its broad inclusion criteria, using explicit methods which limit bias, and assist in revealing new factors. However, we should acknowledge the limitations of our systematic review—using only one data base, not exploring Grey literature (opinions, case studies, and doctoral theses), and excluding studies that were published in other languages.

Unfortunately, there is still insufficient data on the links between genes and depression.

Despite the reported genetic associations, most studies were lacking in statistical power analysis, research samples were small, and most gene polymorphisms have been confirmed in only one study.

Further genetic research with larger research samples is needed to ascertain whether the relationship is random or causal.

## Supplementary Materials

The following supporting information can be downloaded at: https://www.mdpi.com/article/10.3390/medicina58020285/s1, Table S1: Main characteristics and findings of studies included in the systematic review [76,77,78,79,80,81,82,83,84,85,86,87,88,89,90,91,92,93,94,95,96,97,98,99,100,101,102,103,104,105,106,107,108,109,110,111,112,113,114,115,116,117,118,119,120,121,122,123,124,125,126,127,128,129,130,131,132,133,134,135,136,137,138,139,140,141,142,143,144,145,146,147,148,149,150,151,152,153,154,155,156,157,158,159,160,161,162,163,164,165,166,167,168,169,170,171,172]; Table S2: Summary of genotyping and statistical methods used in the eligible studies; Supplementary information, Section A: Risk of bias evaluation using the Tool to Assess Risk of Bias in Case Control Studies; Supplementary information, Section B: Gene functions.

## References

[1] Watanabe S.Y., Iga J.I., Ishii K., Numata S., Shimodera S., Fujita H., Ohmori T. (2015). Biological tests for major depressive disorder that involve leukocyte gene expression assays. J. Psychiatr. Res..

[2] James S.L., Abate D., Abate K.H., Abay S.M., Abbafati C., Abbasi N., Abbastabar H., Abd-Allah F., Abdela J., Abdelalim A. (2018). Global, regional, and national incidence, prevalence, and years lived with disability for 354 diseases and injuries for 195 countries and territories, 1990–2017: A systematic analysis for the Global burden of disease study 2017. Lancet.

[3] Cipriani A., Furukawa T.A., Salanti G., Chaimani A., Atkinson L.Z., Ogawa Y., Leucht S., Ruhe H.G., Turner E.H., Higgins J.P.T. (2018). Comparative efficacy and acceptability of 21 antidepressant drugs for the acute treatment of adults with major depressive disorder: A systematic review and network meta-analysis. Lancet.

[4] Flint J., Kendler K.S. (2014). The genetics of major depression. Neuron.

[5] Rosenblat J.D., Cha D.S., Mansur R.B., McIntyre R.S. (2014). Inflamed moods: A review of the interactions between inflammation and mood disorders. Prog. Neuropsychopharmacol. Biol. Psychiatry.

[6] Yang Z., Ma X., Wang Y., Wang J., Xiang B., Wu J., Deng W., Li M., Wang Q., Li T. (2012). Association of APC and REEP5 gene polymorphisms with major depression disorder and treatment response to antidepressants in a Han Chinese population. Gen. Hosp. Psychiatry.

[7] Lohoff F.W. (2010). Overview of the genetics of major depressive disorder. Curr. Psychiatry Rep..

[8] Sullivan P.F., Neale M.C., Kendler K.S. (2000). Genetic epidemiology of major depression: Review and meta-analysis. AJP.

[9] Shadrina M., Bondarenko E.A., Slominsky P.A. (2018). Genetics factors in major depression disease. Front. Psychiatry.

[10] Wray N.R., Ripke S., Mattheisen M., Trzaskowski M., Byrne E.M., Abdellaoui A., Adams M.J., Agerbo E., Air T.M., Andlauer T.M.F. (2018). Genome-wide association analyses identify 44 risk variants and refine the genetic architecture of major depression. Nat. Genet..

[11] Border R., Johnson E.C., Evans L.M., Smolen A., Berley N., Sullivan P.F., Keller M.C. (2019). No support for historical candidate gene or candidate gene-by-interaction hypotheses for major depression across multiple large samples. Am. J. Psychiatry.

[12] Luo X., Stavrakakis N., Penninx B.W., Bosker F.J., Nolen W.A., Boomsma D.I., de Geus E.J., Smit J.H., Snieder H., Nolte I.M. (2016). Does refining the phenotype improve replication rates? A review and replication of candidate gene studies on Major Depressive Disorder and Chronic Major Depressive Disorder. Am. J. Med. Genet..

[13] Chen M.-H., Lin W.-C., Wu H.-J., Cheng C.-M., Li C.-T., Hong C.-J., Tu P.-C., Bai Y.-M., Tsai S.-J., Su T.-P. (2019). Antisuicidal effect, BDNF Val66Met polymorphism, and low-dose ketamine infusion: Reanalysis of adjunctive ketamine study of Taiwanese patients with treatment-resistant depression (AKSTP-TRD). J. Affect. Disord..

[14] Lansche J. Tool to Assess Risk of Bias in Case Control Studies. https://www.evidencepartners.com/wp-content/uploads/2021/03/Tool-to-Assess-Risk-of-Bias-in-Case-Control-Studies-DistillerSR.pdf..

[15] Li W., Ju K., Li Z., He K., Chen J., Wang Q., Yang B., An L., Feng G., Sun W. (2016). Significant association of GRM7 and GRM8 genes with schizophrenia and major depressive disorder in the Han Chinese population. Eur. Neuropsychopharmacol..

[16] Li H., Wang Y.J., Hua L., Yang Y.T., Zhang M., Zhang D., Wang C., Xu Z.Q.D. (2013). Lack of association between dendritic cell nuclear protein-1 gene and major depressive disorder in the Han Chinese population. Prog. Neuro-Psychopharmacol. Biol. Psychiatry.

[17] Sarubin N., Hilbert S., Naumann F., Zill P., Wimmer A.-M., Nothdurfter C., Rupprecht R., Baghai T.C., Bühner M., Schüle C. (2017). The sex-dependent role of the glucocorticoid receptor in depression: Variations in the NR3C1 gene are associated with major depressive disorder in women but not in men. Eur. Arch. Psychiatry Clin. Neurosci..

[18] Wang L., Shi C., Zhang K., Xu Q. (2014). The gender-specific association of EHD3 polymorphisms with major depressive disorder. Neurosci. Lett..

[19] Liu J., Zhang H.X., Li Z.Q., Li T., Li J.-Y., Wang T., Li Y., Feng G.-Y., Shi Y.-Y., He L. (2017). The YWHAE gene confers risk to major depressive disorder in the male group of Chinese Han population. Prog. Neuro-Psychopharmacol. Biol. Psychiatry.

[20] Santos M., Carvalho S., Lima L., Nogueira A., Assis J., Mota-Pereira J., Pimentel P., Maia D., Correia D., Gomes S. (2014). Common genetic polymorphisms in the ABCB1 gene are associated with risk of major depressive disorder in male Portuguese individuals. Genet. Test. Mol. Biomark..

[21] Zhang J., Chen L., Ma J., Qiao Z., Zhao M., Qi D., Zhao Y., Ban B., Zhu X., He J. (2017). Interaction of estrogen receptor β and negative life events in susceptibility to major depressive disorder in a Chinese Han female population. J. Affect. Disord..

[22] Pérez-Olmos I., Bustamante D., Ibáñez-Pinilla M. (2016). Serotonin transporter gene (5-HTT) polymorphism and major depressive disorder in patients in Bogotá, Colombia. Biomedica.

[23] Du T., Rao S., Wu L., Ye N., Liu Z., Hu H., Xiu J., Shen Y., Xu Q. (2015). An association study of the m6A genes with major depressive disorder in Chinese Han population. J. Affect. Disord..

[24] Nazree N.E., Loke A.C., Zainal N.Z., Mohamed Z. (2015). Lack of association between TPH2 gene polymorphisms with major depressive disorder in multiethnic Malaysian population: Pharmacogenetics of MDD. Asia-Pac. Psychiatry.

[25] Wang L., Chen J., Li Z., Sun W., Chen B., Li S., Li W., Lu D., Wang Y., Shi Y. (2018). Association study of NDST3 gene for schizophrenia, bipolar disorder, major depressive disorder in the Han Chinese population. Am. J. Med. Genet..

[26] Ninomiya-Baba M., Matsuo J., Sasayama D., Hori H., Teraishi T., Ota M., Hattori K., Noda T., Ishida I., Shibata S. (2017). Association of body mass index-related single nucleotide polymorphisms with psychiatric disease and memory performance in a Japanese population. Acta Neuropsychiatr..

[27] Khan R.A.W., Chen J., Wang M., Wen Z., Shen J., Song Z., Li Z., Wang Q., Li W., Xu Y. (2016). Analysis of association between common variants in the SLCO6A1 gene with schizophrenia, bipolar disorder and major depressive disorder in the Han Chinese population. World J. Biol. Psychiatry.

[28] Ma X.-C., Chen C., Zhu F., Jia W., Gao C.-G. (2013). Association of the GDNF gene with depression and heroin dependence, but not schizophrenia, in a Chinese population. Psychiatry Res..

[29] Crisafulli C., Chiesa A., Han C., Lee S.-J., Balzarro B., Andrisano C., Sidoti A., Patkar A.A., Pae C.-U., Serretti A. (2013). Case-control association study of 36 single-nucleotide polymorphisms within 10 candidate genes for major depression and bipolar disorder. Psychiatry Res..

[30] Wang Q., He K., Li Z., Chen J., Li W., Wen Z., Shen J., Qiang Y., Ji J., Wang Y. (2014). The CMYA5 gene confers risk for both schizophrenia and major depressive disorder in the Han Chinese population. World J. Biol. Psychiatry.

[31] Ji W., Li T., Pan Y., Tao H., Ju K., Wen Z., Fu Y., An Z., Zhao Q., Wang T. (2013). CNTNAP2 is significantly associated with schizophrenia and major depression in the Han Chinese population. Psychiatry Res..

[32] Koido K., Traks T., Balõtšev R., Eller T., Must A., Koks S., Maron E., Tõru I., Shlik J., Vasar V. (2012). Associations between LSAMP gene polymorphisms and major depressive disorder and panic disorder. Transl. Psychiatry.

[33] Wang Y., Liu X., Yu Y., Han Y., Wei J., Collier D., Li T., Ma X. (2012). The role of single nucleotide polymorphism of D2 dopamine receptor gene on major depressive disorder and response to antidepressant treatment. Psychiatry Res..

[34] Pereira P.A., Bicalho M.A.C., de Moraes E.N., Malloy-Diniz L., Bozzi I.C.R.S., Nicolato R., Valadão D.R., Miranda D.M., Romano-Silva M.A. (2014). Genetic variant of AKT1 and AKTIP associated with late-onset depression in a Brazilian population: Variant of AKT1 and AKTIP associated with LOD. Int. J. Geriatr. Psychiatry.

[35] Ching-López A., Cervilla J., Rivera M., Molina E., McKenney K., Ruiz I., Rodríguez-Barranco M., Gutiérrez B. (2015). Epidemiological support for genetic variability at hypothalamic-pituitary-adrenal axis and serotonergic system as risk factors for major depression. NDT.

[36] Marsden W.N. (2011). Stressor-induced NMDAR dysfunction as a unifying hypothesis for the aetiology, pathogenesis and comorbidity of clinical depression. Med. Hypotheses.

[37] Ren D., Bi Y., Xu F., Niu W., Zhang R., Hu J., Guo Z., Wu X., Cao Y., Huang X. (2017). Common variants in GRIK4 and major depressive disorder: An association study in the Chinese Han population. Neurosci. Lett..

[38] Dadkhah T., Rahimi-Aliabadi S., Jamshidi J., Ghaedi H., Taghavi S., Shokraeian P., Akhavan-Niaki H., Tafakhori A., Ohadi M., Darvish H. (2017). A genetic variant in miRNA binding site of glutamate receptor 4, metabotropic (GRM4) is associated with increased risk of major depressive disorder. J. Affect. Disord..

[39] Yin H., Pantazatos S.P., Galfalvy H., Huang Y., Rosoklija G.B., Dwork A.J., Burke A., Arango V., Oquendo M.A., Mann J.J. (2016). A pilot integrative genomics study of GABA and glutamate neurotransmitter systems in suicide, suicidal behavior, and major depressive disorder. Am. J. Med. Genet..

[40] Heilig M., Zachrisson O., Thorsell A., Ehnvall A., Mottagui-Tabar S., Sjögren M., Åsberg M., Ekman R., Wahlestedt C., Ågren H. (2004). Decreased cerebrospinal fluid neuropeptide Y (NPY) in patients with treatment refractory unipolar major depression: Preliminary evidence for association with preproNPY gene polymorphism. J. Psychiatr. Res..

[41] Kloster E., Saft C., Akkad D.A., Epplen J.T., Arning L. (2014). Association of age at onset in Huntington disease with functional promoter variations in NPY and NPY2R. J. Mol. Med..

[42] Treutlein J., Strohmaier J., Frank J., Witt S.H., Rietschel L., Forstner A.J., Lang M., Degenhardt F., Dukal H., Herms S. (2017). Association between neuropeptide Y receptor Y2 promoter variant rs6857715 and major depressive disorder. Psychiatr. Genet..

[43] Clarke H., Flint J., Attwood A.S., Munafò M.R. (2010). Association of the 5-HTTLPR genotype and unipolar depression: A meta-analysis. Psychol. Med..

[44] Manoharan A., Shewade D.G., Rajkumar R.P., Adithan S. (2016). Serotonin transporter gene (SLC6A4) polymorphisms are associated with response to fluoxetine in south Indian major depressive disorder patients. Eur. J. Clin. Pharmacol..

[45] Fan T., Hu Y., Xin J., Zhao M., Wang J. (2020). Analyzing the genes and pathways related to major depressive disorder via a systems biology approach. Brain Behav..

[46] Sun N. (2016). Effects of polymorphisms of serotonin transporter promoter (5-HTTLPR) and brain derived neurotrophic factor gene (G196A rs6265) on the risk of major depressive disorder in the Chinese Han population. Eur. Rev. Med. Pharmacol. Sci..

[47] Wang Y., Sun N., Liu Z., Li X., Yang C., Zhang K. (2016). Psychosocial mechanisms of serotonin transporter’s genetic polymorphism in susceptibility to major depressive disorder: Mediated by trait coping styles and interacted with life events. Am. J. Transl. Res..

[48] Kostic M., Canu E., Agosta F., Munjiza A., Novakovic I., Dobricic V., Maria Ferraro P., Miler Jerkovic V., Pekmezovic T., Lecic Tosevski D. (2016). The cumulative effect of genetic polymorphisms on depression and brain structural integrity: Multigene effect and brain integrity in MDD. Hum. Brain Mapp..

[49] Ho P.-S., Ho K.K.-J., Huang W.-S., Yen C.-H., Shih M.-C., Shen L.-H., Ma K.-H., Huang S.-Y. (2013). Association study of serotonin transporter availability and SLC6A4 gene polymorphisms in patients with major depression. Psychiatry Res. Neuroimaging.

[50] Kitzlerová E., Fišar Z., Lelková P., Jirák R., Zvěřová M., Hroudová J., Manukyan A., Martásek P., Raboch J. (2018). Interactions among polymorphisms of susceptibility loci for alzheimer’s disease or depressive disorder. Med. Sci. Monit..

[51] Rao S., Leung C.S.T., Lam M.H., Wing Y.K., Waye M.M.Y., Tsui S.K.W. (2017). Resequencing three candidate genes discovers seven potentially deleterious variants susceptibility to major depressive disorder and suicide attempts in Chinese. Gene.

[52] Stacey D., Cohen-Woods S., Toben C., Arolt V., Dannlowski U., Baune B.T. (2013). Evidence of increased risk for major depressive disorder in individuals homozygous for the high-expressing 5-HTTLPR/rs25531 (LA) allele of the serotonin transporter promoter. Psychiatr. Genet..

[53] Sarmiento-Hernández E.I., Ulloa-Flores R.E., Camarena-Medellín B., Sanabrais-Jiménez M.A., Aguilar-García A., Hernández-Muñoz S. (2019). Association between 5-HTTLPR polymorphism, suicide attempt and comorbidity in Mexican adolescents with major depressive disorder. Actas Esp. Psiquiatr..

[54] Watanabe S.Y., Iga J.I., Numata S., Umehara H., Nishi A., Kinoshita M., Inoshita M., Ohmori T. (2015). Polymorphism in the promoter of the gene for the serotonin transporter affects the age of onset of major depressive disorder in the Japanese population. J. Affect. Disord..

[55] Han K.M., Choi S., Kim A., Kang J., Won E., Tae W.-S., Kim Y.-K., Lee M.-S., Ham B.-J. (2018). The effects of 5-HTTLPR and BDNF Val66Met polymorphisms on neurostructural changes in major depressive disorder. Psychiatry Res. Neuroimaging.

[56] Tatham E.L., Ramasubbu R., Gaxiola-Valdez I., Cortese F., Clark D., Goodyear B., Foster J., Hall G.B. (2016). White matter integrity in major depressive disorder: Implications of childhood trauma, 5-HTTLPR and BDNF polymorphisms. Psychiatry Res. Neuroimaging.

[57] Kalska H., Pesonen U., Lehikoinen S., Stenberg J.-H., Lipsanen J., Niemi-Pynttäri J., Tuunainen A. (2013). Association between neurocognitive impairment and the short allele of the 5-HTT promoter polymorphism in depression: A pilot study. Psychiatry J..

[58] Ahdidan J., Foldager L., Rosenberg R., Rodell A., Videbech P., Mors O. (2013). Hippocampal volume and serotonin transporter polymorphism in major depressive disorder. Acta Neuropsychiatr..

[59] Bi Y., Huang X., Niu W., Chen S., Wu X., Cao Y., Zhang R., Yang F., Wang L., Li W. (2017). Common variants in SLC6A2, SLC6A3, DRD2, and major depressive disorder: An association study in the Chinese Han population. Psychiatr. Genet..

[60] Wang Y., Sun N., Li S., Du Q., Xu Y., Liu Z., Zhang K. (2015). A genetic susceptibility mechanism for major depression: Combinations of polymorphisms defined the risk of major depression and subpopulations. Medicine.

[61] Cao S.X., Li H.F., Zhao X.F., Pang J.Y., Liu Q., Xie G.R. (2018). Association between T-182C, G1287A polymorphism in NET gene and suicidality in major depressive disorder in Chinese patients. Int. J. Psychiatry Clin. Pract..

[62] Drago A., Crisafulli C., Sidoti A., Serretti A. (2011). The molecular interaction between the glutamatergic, noradrenergic, dopaminergic and serotoninergic systems informs a detailed genetic perspective on depressive phenotypes. Prog. Neurobiol..

[63] Yang C., Sun N., Ren Y., Sun Y., Xu Y., Li A., Wu K., Zhang K. (2012). Association between AKT1 gene polymorphisms and depressive symptoms in the Chinese Han population with major depressive disorder. Neural Regen. Res..

[64] Shishkina T.V., Mishchenko T.A., Mitroshina E.V., Shirokova O.M., Pimashkin A.S., Kastalskiy I.A., Mukhina I.V., Kazantsev V.B., Vedunova M.V. (2018). Glial cell line-derived neurotrophic factor (GDNF) counteracts hypoxic damage to hippocampal neural network function in vitro. Brain Res..

[65] Tsybko A.S., Ilchibaeva T.V., Popova N.K. (2017). Role of glial cell line-derived neurotrophic factor in the pathogenesis and treatment of mood disorders. Rev. Neurosci..

[66] Mackenzie F., Ruhrberg C. (2012). Diverse roles for VEGF-A in the nervous system. Development.

[67] Uribe E., Wix R. (2012). Neuronal migration, apoptosis and bipolar disorder. Rev. Psiquiatr. Salud Ment..

[68] Prata D.P., Breen G., Osborne S., Munro J., Clair D.S., Collier D.A. (2009). An association study of the neuregulin 1 gene, bipolar affective disorder and psychosis. Psychiatr. Genet..

[69] Buttenschøn H.N., Demontis D., Kaas M., Elfving B., Mølgaard S., Gustafsen C., Kaerlev L., Petersen C.M., Børglum A.D., Mors O. (2015). Increased serum levels of sortilin are associated with depression and correlated with BDNF and VEGF. Transl. Psychiatry.

[70] Khalilova Z.L., Zainullina A.G., Valiullina A.R., Zakharova G.G., Valinurov R.G., Khusnutdinova E.K. (2013). Association of YWHAE gene polymorphism with suicidal behavior. Russ. J. Genet..

[71] Gałecka E., Szemraj J., Bieńkiewicz M., Majsterek I., Przybyłowska-Sygut K., Gałecki P., Lewiński A. (2013). Single nucleotide polymorphisms of NR3C1 gene and recurrent depressive disorder in population of Poland. Mol. Biol. Rep..

[72] Gałecki P., Gałecka E., Maes M., Orzechowska A., Berent D., Talarowska M., Bobińska K., Lewiński A., Bieńkiewicz M., Szemraj J. (2013). Vascular endothelial growth factor gene (VEGFA) polymorphisms may serve as prognostic factors for recurrent depressive disorder development. Prog. Neuro-Psychopharmacol. Biol. Psychiatry.

[73] Li Z., He B., Xu J., Dai N., Ping L., Zhou C., Shen Z., Xu X., Cheng Y. (2020). Roles of 5,10-methylenetetrahydrofolate reductase C677T polymorphisms in first-episode, drug-naive adult patients with depression. Front. Psychiatry.

[74] Zhang C., Ran L., Ai M., Wang W., Chen J., Wu T., Liu W., Jin J., Wang S., Kuang L. (2020). Targeted sequencing of the bdnf gene in young chinese han people with major depressive disorder. Mol. Genet. Genom. Med..

[75] Fratelli C., Siqueira J., Silva C., Ferreira E., Silva I. (2020). 5HTTLPR genetic variant and major depressive disorder: A review. Genes.

[76] Wang Q., Wang Y., Ji W., Zhou G., He K., Li Z., Chen J., Li W., Wen Z., Shen J. (2015). SNAP25 is associated with schizophrenia and major depressive disorder in the Han Chinese population. J. Clin. Psychiatry.

[77] Aldoghachi A.F., Tor Y.S., Redzun S.Z., Lokman K.A.B., Razaq N.A.A., Shahbudin A.F., Badamasi I.M., Cheah P.-S., Stanslas J., Veerakumarasivam A. (2019). Screening of brain-derived neurotrophic factor (BDNF) single nucleotide polymorphisms and plasma BDNF levels among Malaysian major depressive disorder patients. Hashimoto K, editor. PLoS ONE.

[78] Chao J.K., Yang M.C., Chen C.S., Wang I.C., Kao W.T., Shi M.D. (2019). A gender-specific COMT haplotype contributes to risk modulation rather than disease severity of major depressive disorder in a Chinese population. J. Affect. Disord..

[79] Yang J., Zhao X., Ma J., Qiao Z., Yang X., Zhao E., Ban B., Zhu X., Cao D., Yang Y. (2019). The interaction of TPH2 and 5-HT2A polymorphisms on major depressive disorder susceptibility in a Chinese Han population: A case-control study. Front. Psychiatry.

[80] Xie T., Stathopoulou M.G., de Andrés F., Siest G., Murray H., Martin M., Cobaleda J., Delgado A., Lamont J., Peñas-LIedó E. (2017). VEGF-related polymorphisms identified by GWAS and risk for major depression. Transl. Psychiatry.

[81] Mahmood S., Evinová A., Škereňová M., Ondrejka I., Lehotský J. (2016). Association of EGF, IGFBP-3 and TP53 gene polymorphisms with major depressive disorder in Slovak population. Cent. Eur. J. Public Health.

[82] Zhang C., Wu Z., Zhao G., Wang F., Fang Y. (2016). Identification of IL6 as a susceptibility gene for major depressive disorder. Sci. Rep..

[83] Sayadi M.A., Achour O., Ezzaher A., Hellara I., Omezzine A., Douki W., Bousslama A., Gaha L., Najjar M.F. (2016). CT genotype of 5,10-methylenetetrahydrofolate reductase (MTHFR) C677T polymorphism is protector factor of major depressive disorder in the Tunisian population: A case control study. Ann. Gen. Psychiatry.

[84] Zhang L., Xie W.W., Wu R.R., Yu Y., Zhao J.-P., Li L.H. (2015). Case-control association study of ABCB1 gene and major depressive disorder in a local Chinese Han population. NDT.

[85] Liang Y., Zhao G., Sun R., Mao Y., Li G., Chen X., Gao L., Hu Z. (2015). Genetic variants in the promoters of let-7 family are associated with an increased risk of major depressive disorder. J. Affect. Disord..

[86] Zhang Z., Ni J., Zhang J., Tang W., Li X., Wu Z., Zhang C. (2016). A haplotype in the 5′-upstream region of the NDUFV2 gene is associated with major depressive disorder in Han Chinese. J. Affect. Disord..

[87] Congiu C., Minelli A., Bonvicini C., Bortolomasi M., Sartori R., Maj C., Scassellati C., Maina G., Trabucchi L., Segala M. (2015). The role of the potassium channel gene KCNK2 in major depressive disorder. Psychiatry Res..

[88] Zhou Y., Wang J., He Y., Zhou J., Xi Q., Song X., Ye Y., Ying B. (2015). Association between dopamine beta-hydroxylase 19-bp insertion/deletion polymorphism and major depressive disorder. J. Mol. Neurosci..

[89] Kokut S., Atay I.M., Uz E., Akpinar A., Demirdas A. (2015). The polymorphisms of Ser49Gly and Gly389Arg in beta-1-adrenergic receptor gene in major depression. Arch. Neuropsychiatr..

[90] McFarquhar M., Elliott R., McKie S., Thomas E., Downey D., Mekli K., Toth Z.G., Anderson I.M., Deakin J.W., Juhasz G. (2014). TOMM40 rs2075650 may represent a new candidate gene for vulnerability to major depressive disorder. Neuropsychopharmacology.

[91] Mocking R.J.T., Lok A., Assies J., Koeter M.W.J., Visser I., Ruhé H.G., Bockting C.L.H., Schene A.H. (2013). Ala54thr fatty acid-binding protein 2 (FABP2) polymorphism in recurrent depression: Associations with fatty acid concentrations and waist circumference. Alquier T, editor. PLoS ONE.

[92] Hua P., Liu W., Chen D., Zhao Y., Chen L., Zhang N., Wang C., Guo S., Wang L., Xiao H. (2014). Cry1 and Tef gene polymorphisms are associated with major depressive disorder in the Chinese population. J. Affect. Disord..

[93] He M., Yan H., Duan Z.-X., Qu W., Gong H.-Y., Fan Z.-L., Kang J.-Y., Li B.-C., Wang J.-M. (2013). Genetic distribution and association analysis of DRD2 gene polymorphisms with major depressive disorder in the Chinese Han population. Int. J. Clin. Exp. Pathol..

[94] Evinova A., Babusikova E., Straka S., Ondrejka I., Lehotsky J. (2012). Analysis of genetic polymorphisms of brain-derived neurotrophic factor and methylenetetrahydrofolate reductase in depressed patients in a Slovak (Caucasian) population. GPB.

[95] Wang Y.J., Li H., Yang Y.T., Tie C.-L., Li F., Xu Z.-Q.D., Wang C.-Y. (2013). Association of galanin and major depressive disorder in the Chinese Han population. PLoS ONE.

[96] Tian W., Zhang J., Zhang K., Yang H., Sun Y., Shen Y., Xu Q. (2012). A study of the functional significance of epidermal growth factor in major depressive disorder. Psychiatr. Genet..

[97] Minelli A., Scassellati C., Cloninger C.R., Tessari E., Bortolomasi M., Bonvicini C., Giacopuzzi M., Frisoni G.B., Gennarelli M. (2012). PCLO gene: Its role in vulnerability to major depressive disorder. J. Affect. Disord..

[98] Vereczkei A., Abdul-Rahman O., Halmai Z., Nagy G., Szekely A., Somogyi A., Faludi G., Nemoda Z. (2019). Association of purinergic receptor P2RX7 gene polymorphisms with depression symptoms. Prog. Neuro-Psychopharmacol. Biol. Psychiatry..

[99] Tao S., Chattun M.R., Yan R., Geng J., Zhu R., Shao J., Lu Q., Yao Z. (2018). TPH-2 gene polymorphism in major depressive disorder patients with early-wakening symptom. Front. Neurosci..

[100] Cao S., Zhao X., Li H. (2012). No association between a polymorphism of the adenylate cyclase type IX gene and major depressive disorder in the Chinese Han population. Neural Regen. Res..

[101] Zeng D., He S., Yu S., Li G., Ma C., Wen Y., Shen Y., Yu Y., Li H. (2018). Analysis of the association of MIR124-1 and its target gene RGS4 polymorphisms with major depressive disorder and antidepressant response. NDT.

[102] Şahin Can M., Baykan H., Baykan Ö., Erensoy N., Karlıdere T. (2017). Vitamin D levels and vitamin D receptor gene polymorphism in major depression. Psychiatr. Danub..

[103] Han K.M., Won E., Kang J., Choi S., Kim A., Lee M.-S., Tae W.-S., Ham B.-J. (2017). TESC gene-regulating genetic variant (rs7294919) affects hippocampal subfield volumes and parahippocampal cingulum white matter integrity in major depressive disorder. J. Psychiatr. Res..

[104] Cribb L., Murphy J., Froud A., Oliver G., Bousman C.A., Ng C.H., Sarris J. (2018). Erythrocyte polyunsaturated fatty acid composition is associated with depression and FADS genotype in Caucasians. Nutr. Neurosci..

[105] Wang Q., Ji W., He K., Li Z., Chen J., Li W., Wen Z., Shen J., Yu Q., Feng G. (2018). Genetic analysis of common variants in the ZNF804A gene with schizophrenia and major depressive disorder. Psychiatr. Genet..

[106] Wang L., Liu Z., Cao X., Li J., Zhang A., Sun N., Yang C., Zhang K. (2017). A combined study of SLC6A15 gene polymorphism and the resting-state functional magnetic resonance imaging in first-episode drug-naive major depressive disorder. Genet. Test. Mol. Biomark..

[107] Won E., Han K.M., Kang J., Kim A., Yoon H.-K., Chang H.S., Park J.-Y., Lee M.-S., Greenberg T., Tae W.-S. (2017). Vesicular monoamine transporter 1 gene polymorphism and white matter integrity in major depressive disorder. Prog. Neuro-Psychopharmacol. Biol. Psychiatry.

[108] Tollenaar M.S., Molendijk M.L., Penninx B.W.J.H., Milaneschi Y., Antypa N. (2017). The association of childhood maltreatment with depression and anxiety is not moderated by the oxytocin receptor gene. Eur. Arch. Psychiatry Clin. Neurosci..

[109] Ma J., Wang L., Yang Y., Qiao Z., Fang D., Qiu X., Yang X., Zhu X., He J., Pan H. (2017). GNB3 and CREB1 gene polymorphisms combined with negative life events increase susceptibility to major depression in a Chinese Han population. PLoS ONE.

[110] Han K.M., Won E., Sim Y., Kang J., Han C., Kim Y.-K., Kim S.-H., Joe S.-H., Lee M.-S., Tae W.-S. (2017). Influence of FKBP5 polymorphism and DNA methylation on structural changes of the brain in major depressive disorder. Sci. Rep..

[111] Mandelli L., Wang S.M., Han C., Lee S.-J., Patkar A.A., Masand P.S., Pae C.-U., Serretti A. (2017). The impact of a single nucleotide polymorphism in SIGMAR1 on depressive symptoms in major depressive disorder and bipolar disorder. Adv. Ther..

[112] Wang Y., Li L., Xu C., Cao X., Liu Z., Sun N., Zhang A., Li X., Zhang K. (2017). Polymorphism of ERK/PTPRR genes in major depressive disorder at resting-state brain function. Dev. Neuropsychol..

[113] Xu C., Yang C., Zhang A., Xu Y., Li X., Liu Z., Liu S., Sun N., Zhang K. (2017). The interaction of miR-34b/c polymorphisms and negative life events increases susceptibility to major depressive disorder in Han Chinese population. Neurosci. Lett..

[114] Han D., Qiao Z., Chen L., Qiu X., Fang D., Yang X., Ma J., Chen M., Yang J., Wang L. (2017). Interactions between the vascular endothelial growth factor gene polymorphism and life events in susceptibility to major depressive disorder in a Chinese population. J. Affect. Disord..

[115] Choi S., Han K.M., Kang J., Won E., Chang H.S., Tae W.S., Son K.R., Kim S.-J., Lee M.-S., Ham B.-J. (2016). Effects of a polymorphism of the neuronal amino acid transporter SLC6A15 gene on structural integrity of white matter tracts in major depressive disorder. PLoS ONE.

[116] Mushtaq R., Tarfarosh S.F.A., Dar M.M., Hussain A., Shoib S., Shah T., Shah S., Manzoor M. (2016). Is there a link between depressive disorders and tryptophan hydroxylase 1 (TPH1) gene polymorphism? Study from a distressed area, Kashmir (india). Cureus.

[117] Bondarenko E., Shadrina M., Grishkina M., Druzhkova T., Akzhigitov R., Gulyaeva N., Guekht A., Slominsky P. (2016). Genetic analysis of BDNF, GNB3, MTHFR, ACE and APOE variants in major and recurrent depressive disorders in Russia. Int. J. Med. Sci..

[118] Sublette M.E., Vaquero C., Baca-Garcia E., Pachano G., Huang Y., Oquendo M.A., Mann J.J. (2016). Lack of association of SNPs from the FADS1-FADS2 gene cluster with major depression or suicidal behavior. Psychiatr. Genet..

[119] Wei Y.B., Martinsson L., Liu J.J., Forsell Y., Schalling M., Backlund L., Lavebratt C. (2016). hTERT genetic variation in depression. J. Affect. Disord..

[120] Ma J., Xiao H., Yang Y., Cao D., Wang L., Yang X., Qiu X., Qiao Z., Song J., Liu Y. (2015). Interaction of tryptophan hydroxylase 2 gene and life events in susceptibility to major depression in a Chinese Han population. J. Affect. Disord..

[121] Wen Z., Chen J., Khan R.A.W., Song Z., Wang M., Li Z., Shen J., Li W., Shi Y. (2016). Genetic association between NRG1 and schizophrenia, major depressive disorder, bipolar disorder in Han Chinese population. Am. J. Med. Genet..

[122] Wen Z., Chen J., Khan R.A.W., Wang M., Song Z., Li Z., Shen J., Li W., Shi Y. (2016). Polymorphisms in NRGN are associated with schizophrenia, major depressive disorder and bipolar disorder in the Han Chinese population. J. Affect. Disord..

[123] Won E., Kang J., Kim A., Choi S., Han K.-M., Tae W.S., Chang H.S., Son K.R., Greenberg T., Joe S.-H. (2016). Influence of BclI C/G (rs41423247) on hippocampal shape and white matter integrity of the parahippocampal cingulum in major depressive disorder. Psychoneuroendocrinology.

[124] Quteineh L., Preisig M., Rivera M., Milaneschi Y., Castelao E., Gholam-Rezaee M., Vandenberghe F., Saigi-Morgui N., Delacrétaz A., Cardinaux J.-R. (2016). Association of CRTC1 polymorphisms with obesity markers in subjects from the general population with lifetime depression. J. Affect. Disord..

[125] Chang H.S., Won E.S., Lee H.Y., Ham B.J., Kim Y.G., Lee M.S. (2015). Association of ARRB1 polymorphisms with the risk of major depressive disorder and with treatment response to mirtazapine. J. Psychopharmacol..

[126] He M., He H., Yang L., Zhang J., Chen K., Duan Z. (2019). Functional tag SNPs inside the DRD2 gene as a genetic risk factor for major depressive disorder in the Chinese Han population. Int. J. Clin. Exp. Pathol..

[127] Milaneschi Y., Lamers F., Mbarek H., Hottenga J.J., Boomsma D.I., Penninx B.W.J.H. (2014). The effect of FTO rs9939609 on major depression differs across MDD subtypes. Mol. Psychiatry.

[128] Chang H.S., Won E., Lee H.Y., Ham B.J., Lee M.S. (2015). Association analysis for corticotropin releasing hormone polymorphisms with the risk of major depressive disorder and the response to antidepressants. Behav. Brain Res..

[129] Gabriela Nielsen M., Congiu C., Bortolomasi M., Bonvicini C., Bignotti S., Abate M., Milanesi E., Conca A., Cattane N., Tessari E. (2015). MTHFR: Genetic variants, expression analysis and COMT interaction in major depressive disorder. J. Affect. Disord..

[130] Inoue A., Akiyoshi J., Muronaga M., Masuda K., Aizawa S., Hirakawa H., Ishitobi Y., Higuma H., Maruyama Y., Ninomiya T. (2015). Association of TMEM132D, COMT, and GABRA6 genotypes with cingulate, frontal cortex and hippocampal emotional processing in panic and major depressive disorder. Int. J. Psychiatry Clin. Pract..

[131] Traks T., Koido K., Balõtšev R., Eller T., Kõks S., Maron E., Tõru I., Shlik J., Vasar E., Vasar V. (2015). Polymorphisms of IKBKE gene are associated with major depressive disorder and panic disorder. Brain Behav..

[132] Li W., Ji W., Li Z., He K., Wang Q., Chen J., Qiang Y., Feng G., Li X., Shen J. (2015). Genetic association of ACSM1 variation with schizophrenia and major depressive disorder in the Han Chinese population. Am. J. Med. Genet..

[133] Lee S.M., Lee S., Kang W.S., Jahng G.-H., Park H.J., Kim S.K., Park J.K. (2018). Gray matter volume reductions were associated with TPH1 polymorphisms in depressive disorder patients with suicidal attempts. Psychiatry Investig..

[134] Elfving B., Buttenschøn H.N., Foldager L., Poulsen P.H.P., Andersen J.H., Grynderup M.B., Hansen Å.M., Kolstad H.A., Kaerlev L., Mikkelsen S. (2012). Depression, the Val66Met polymorphism, age, and gender influence the serum BDNF level. J. Psychiatr. Res..

[135] Jia W., Zhang R., Wu B., Dai Z., Zhu Y., Li P., Zhu F. (2014). Metabotropic glutamate receptor 3 is associated with heroin dependence but not depression or schizophrenia in a chinese population. PLoS ONE.

[136] Sasayama D., Hori H., Yamamoto N., Nakamura S., Teraishi T., Tatsumi M., Hattori K., Ota M., Higuchi T., Kunugi H. (2014). ITIH3 polymorphism may confer susceptibility to psychiatric disorders by altering the expression levels of GLT8D1. J. Psychiatr. Res..

[137] Wang X., Wang Z., Wu Y., Yuan Y., Hou Z., Hou G. (2014). Association analysis of the catechol-O-methyltransferase /methylenetetrahydrofolate reductase genes and cognition in late-onset depression. Psychiatry Clin. Neurosci..

[138] Zhang C., Wu Z., Hong W., Wang Z., Peng D., Chen J., Yuan C., Yu S., Xu L., Fang Y. (2014). Influence of BCL2 gene in major depression susceptibility and antidepressant treatment outcome. J. Affect. Disord..

[139] Stacey D., Redlich R., Opel N., Grotegerd D., Arolt V., Kugel H., Heindel W., Baune B.T., Dannlowski U. (2014). No evidence of DISC1-associated morphological changes in the hippocampus, anterior cingulate cortex, or striatum in major depressive disorder cases and healthy controls. J. Affect. Disord..

[140] Frazier T.W., Youngstrom E.A., Frankel B.A., Zunta-Soares G.B., Sanches M., Escamilla M., Nielsen D.A., Soares J.C. (2014). Candidate gene associations with mood disorder, cognitive vulnerability, and fronto-limbic volumes. Brain Behav..

[141] Ozbey G., Yucel B., Taycan S.E., Kan D., Bodur N.E., Arslan T., Percin F., Yuksel N., Guzey C., Uluoglu C. (2014). ABCB1 C3435T polymorphism is associated with susceptibility to major depression, but not with a clinical response to citalopram in a Turkish population. Pharmacol. Rep..

[142] Szczepankiewicz A., Leszczyńska-Rodziewicz A., Pawlak J., Narozna B., Rajewska-Rager A., Wilkosc M., Zaremba D., Maciukiewicz M., Twarowska-Hauser J. (2014). FKBP5 polymorphism is associated with major depression but not with bipolar disorder. J. Affect. Disord..

[143] Shen X., Wu Y., Guan T., Wang X., Qian M., Lin M., Shen Z., Sun J., Zhong H., Yang J. (2014). Association analysis of COMT/MTHFR polymorphisms and major depressive disorder in Chinese Han population. J. Affect. Disord..

[144] Elbozan Cumurcu B., Ozyurt H., Ates O., Gogcegoz Gul I., Demir S., Karlıdag R. (2013). Analysis of manganese superoxide dismutase (MnSOD: Ala-9Val) and glutathione peroxidase (GSH-Px: Pro 197 Leu) gene polymorphisms in mood disorders. Bosn. J. Basic Med. Sci..

[145] Halmai Z., Dome P., Vereczkei A., Abdul-Rahman O., Szekely A., Gonda X., Faludi G., Sasvari-Szekely M., Nemoda Z. (2013). Associations between depression severity and purinergic receptor P2RX7 gene polymorphisms. J. Affect. Disord..

[146] Mitjans M., Serretti A., Fabbri C., Gastó C., Catalán R., Fañanás L., Arias B. (2013). Screening genetic variability at the CNR1 gene in both major depression etiology and clinical response to citalopram treatment. Psychopharmacology.

[147] Chen J., Xu Y., Zhang J., Liu Z., Xu C., Zhang K., Shen Y., Xu Q. (2012). Genotypic association of the DAOA gene with resting-state brain activity in major depression. Mol. Neurobiol..

[148] Firouzabadi N., Shafiei M., Bahramali E., Ebrahimi S.A., Bakhshandeh H., Tajik N. (2012). Association of angiotensin-converting enzyme (ACE) gene polymorphism with elevated serum ACE activity and major depression in an Iranian population. Psychiatry Res..

[149] Carballedo A., Amico F., Ugwu I., Fagan A.J., Fahey C., Morris D., Meaney J.F., Leemans A., Frodl T. (2012). Reduced fractional anisotropy in the uncinate fasciculus in patients with major depression carrying the met-allele of the Val66Met brain-derived neurotrophic factor genotype. Am. J. Med. Genet..

[150] Green E.K., Grozeva D., Forty L., Gordon-Smith K., Russell E., Farmer A., Hamshere M., Jones I.R., Jones L., McGuffin P. (2013). Association at SYNE1 in both bipolar disorder and recurrent major depression. Mol. Psychiatry.

[151] Wigner P., Czarny P., Synowiec E., Bijak M., Białek K., Talarowska M., Galecki P., Szemraj J., Sliwinski T. (2018). Variation of genes involved in oxidative and nitrosative stresses in depression. Eur. Psychiatr..

[152] Wigner P., Czarny P., Synowiec E., Bijak M., Białek K., Talarowska M., Galecki P., Szemraj J., Sliwinski T. (2018). Association between single nucleotide polymorphisms of TPH1 and TPH2 genes, and depressive disorders. J. Cell. Mol. Med..

[153] Czarny P., Wigner P., Strycharz J., Watala C., Swiderska E., Synowiec E., Galecki P., Talarowska M., Szemraj J., Su K.-P. (2018). Single-nucleotide polymorphisms of uracil-processing genes affect the occurrence and the onset of recurrent depressive disorder. PeerJ.

[154] Hu Y., Hong W., Smith A., Yu S., Li Z., Wang D., Yuan C., Cao L., Wu Z., Huang J. (2017). Association analysis between mitogen-activated protein/extracellular signal-regulated kinase (MEK) gene polymorphisms and depressive disorder in the Han Chinese population. J. Affect. Disord..

[155] Gałecka E., Talarowska M., Orzechowska A., Górski P., Bieńkiewicz M., Szemraj J. (2015). Association of the DIO2 gene single nucleotide polymorphisms with recurrent depressive disorder. Acta Biochim. Pol..

[156] Czarny P., Kwiatkowski D., Galecki P., Talarowska M., Orzechowska A., Bobinska K., Bielecka-Kowalska A., Szemraj J., Maes M., Su K.-P. (2015). Association between single nucleotide polymorphisms of MUTYH, hOGG1 and NEIL1 genes, and depression. J. Affect. Disord..

[157] Seripa D., Panza F., D’Onofrio G., Paroni G., Bizzarro A., Fontana A., Paris F., Cascavilla L., Copetti M., Masullo C. (2013). The serotonin transporter gene locus in late-life major depressive disorder. Am. J. Geriatr. Psychiatry.

[158] Bobińska K., Szemraj J., Czarny P., Gałecki P. (2016). Role of MMP-2, MMP-7, MMP-9 and TIMP-2 in the development of recurrent depressive disorder. J. Affect. Disord..

[159] Gałecka E., Talarowska M., Maes M., Su K.P., Górski P., Szemraj J. (2016). Polymorphisms of iodothyronine deiodinases (DIO1, DIO3) genes are not associated with recurrent depressive disorder. Pharmacol. Rep..

[160] Wigner P., Czarny P., Synowiec E., Bijak M., Talarowska M., Galecki P., Szemraj J., Sliwinski T. (2018). Variation of genes encoding KAT1, AADAT and IDO1 as a potential risk of depression development. Eur. Psychiatr..

[161] Taylor W.D., Zhao Z., Ashley-Koch A., Payne M.E., Steffens D.C., Krishnan R.R., Hauser E., MacFall J.R. (2013). Fiber tract-specific white matter lesion severity findings in late-life depression and by AGTR1 A1166C genotype. Hum. Brain Mapp..

[162] Wang P., Yang Y., Yang X., Qiu X., Qiao Z., Wang L., Zhu X., Sui H., Ma J. (2015). CREB1 gene polymorphisms combined with environmental risk factors increase susceptibility to major depressive disorder (MDD). Int. J. Clin. Exp. Pathol..

[163] He Y., Zhou Y., Xi Q., Cui H., Luo T., Song H., Nie X., Wang L., Ying B. (2012). Genetic variations in microRNA processing genes are associated with susceptibility in depression. DNA Cell Biol..

[164] Buttenschøn H.N., Krogh J., Nielsen M.N., Kaerlev L., Nordentoft M., Mors O. (2017). Association analyses of depression and genes in the hypothalamus-pituitary-adrenal axis. Acta Neuropsychiatr..

[165] Yuan F., Yuan R., Niu W., Ren D., Bi Y., Xu F., Hu J., Sun Q., Ma G., Guo Z. (2018). No association of NR3C1 polymorphisms with major depressive disorder in the Chinese Han population. Psychiatr. Genet..

[166] Froud A., Murphy J., Cribb L., Ng C.H., Sarris J. (2019). The relationship between dietary quality, serum brain-derived neurotrophic factor (BDNF) level, and the Val66met polymorphism in predicting depression. Nutr. Neurosci..

[167] Czarny P., Kwiatkowski D., Toma M., Gałecki P., Orzechowska A., Bobińska K., Bielecka-Kowalska A., Szemraj J., Berk M., Anderson G. (2016). Single-nucleotide polymorphisms of genes involved in repair of oxidative DNA damage and the risk of recurrent depressive disorder. Med. Sci. Monit..

[168] Gałecki P., Orzechowska A., Berent D., Talarowska M., Bobińska K., Gałecka E., Lewiński A., Maes M., Szemraj J. (2013). Vascular endothelial growth factor receptor 2 gene (KDR) polymorphisms and expression levels in depressive disorder. J. Affect. Disord..

[169] Wei Y., Bu S., Liu X., Li H. (2015). Association study of three single-nucleotide polymorphisms in the cyclic adenosine monophosphate response element binding 1 gene and major depressive disorder. Exp. Ther. Med..

[170] Mihailova S., Ivanova-Genova E., Lukanov T., Stoyanova V., Milanova V., Naumova E. (2016). A study of TNF-α, TGF-β, IL-10, IL-6, and IFN-γ gene polymorphisms in patients with depression. J. Neuroimmunol..

[171] Hayashi K., Yoshimura R., Kakeda S., Kishi T., Abe O., Umene-Nakano W., Katsuki A., Hori H., Ikenouchi-Sugita A., Watanabe K. (2014). COMT Val158Met, but not BDNF Val66Met, is associated with white matter abnormalities of the temporal lobe in patients with first-episode, treatment-naive major depressive disorder: A diffusion tensor imaging study. NDT.

[172] Li T., Zeng Z., Zhao Q., Wang T., Huang K., Li J., Li Y., Liu J., Wei Z., Wang Y. (2013). FoxP2 is significantly associated with schizophrenia and major depression in the Chinese Han population. World J. Biol. Psychiatry.

