# Supplementary material for: A Systematic Review of Candidate Genes for Major Depression
Source: Medicina (Kaunas). 2022 Feb 14;58(2):285. doi: 10.3390/medicina58020285 (PMC8875554; doi:10.3390/medicina58020285)
Supplement: Supplementary file 1 [file medicina-58-00285-s001.zip › Supplementary information, Section A.pdf]

Supplementary information, Section A . Risk of bias evaluation using the "Tool to Assess Risk of Bias in Case Control Studies"

Risk of publication bias assessment process

Although according to our protocol, initially we planned to use a Cochrane risk of bias tool, however after careful analysis, it became clear, that it was not suitable for case-control studies, which were exclusively included in our review. That is why, we decided to use a different tool, which is recommended by CLARITY group for case-control studies, the "Tool to Assess Risk of Bias in Case Control Studies" [14].

2 researchers screened eligible studies according to the tool. Each study was assessed in 5 dimensions: (i) assessment of exposure, (ii) assessment of outcome development, (iii) case group subject selection, (iv) control group subject selection, (v) group comparability, adjustment to prognostic factors. Then each study was appointed to one of the three categories: of low, higher or high risk of bias based on each dimension. Afterwards studies were stratified into 3 groups of overall low, higher or high risk of bias. During the process of assessment, it became clear, that in some cases, comprehensive information could not be obtained from the original articles, in those cases due to lack of important data, the studies were appointed into a separate group of unclear risk of bias. You can see the evaluation of each study in the Supplementary information, Appendix B.

Author (ref)	doi	1	2	3	4	5	Overall risk of bias
Yang J, et al.	10.3389/fpsyt.2019.00172	low	low	low	low	low	low
Aldoghachi AF et al.	10.1371/journal.pone.0211241	low	low	low	low	low	low
Chao JK et al.	10.1016/j.jad.2018.12.088	low	low	low	low	high	high
Decheng Ren et al.	10.1016/j.neulet.2017.05.071	low	low	low	low	low	low
Jie Liu et al.	10.1016/j.pnpbp.2017.04.013	low	low	low	low	higher	higher
T Xie et al.	10.1038/tp.2017.36	low	low	low	low	low	low
Yan Bia et al.	10.1097/YPG.0000000000000163	low	low	low	low	high	high
Tahereh Dadkhah et al.	10.1016/j.jad.2016.10.008	low	low	low	low	low	low
Silvia Mahmood et al.	10.21101/cejph.a4301	low	low	low	low	unclear	unclear
Isabel Pérez-Olmos et al.	10.7705/biomedica.v36i3.3014	low	low	low	low	low	
Jens Treutlein et al.	10.1097/YPG.0000000000000149	low	low	low	low	high	low
Nina Sarubin et al.	10.1007/s00406-016-0722-5	low	low	low	low	low	high
Chen Zhang et al.	10.1038/srep31264	low	low	low	low	low	low
Mohamed Amine Sayadi et al.	10.1186/s12991-016-0103-5	low	low	low	low	unclear	low
N. SUN et al.	Eur Rev Med Pharmacol Sci. 2016 May;20(9):1852-9.	low	low	low	low	unclear	unclear
Zaifu Zhang et al.	10.1016/j.jad.2015.10.034	low	low	low	low	low	unclear
Wei-Wei Xie et al.	10.2147/NDT.S87175. eCollection 2015	low	low	low	low	low	low
YANGE WEI et al.	10.3892/etm.2015.2408	low	low	low	low	unclear	low
Yanfang Wang et al.	10.1097/MD.0000000000000778	low	low	low	low	low	unclear
Yundan Liang et al.	10.1016/j.jad.2015.04.035	low	low	low	low	low	low
Tingfu Du et al.	10.1016/j.jad.2015.05.025	low	low	low	low	high	low
Süleyman Kokut et al.	10.5152/npa.2015.7321	low	low	low	low	unclear	high
Shinya Watanabe et al.	10.1016/j.jad.2015.05.009	low	low	low	low	high	unclear
Chiara Congiu et al.	10.1016/j.psychres.2014.11.061	low	low	low	low	low	high
Yi Zhou et al.	10.1007/s12031-014-0339-y	low	low	low	low	low	low
Ping Hua et al.	10.1016/j.jad.2013.11.019	low	low	low	low	unclear	low
Xingwang Li et al.	10.1007/s10519-014-9645-y	low	low	low	low	low	unclear
Martyn McFarquhar et al.	10.1038/npp.2014.22	low	high	low	low	low	low
Nur Elia Nazree BSc et al.	10.1111/appy.12118	low	low	low	low	high	low
Roel J. T. Mocking et al.	10.1371/journal.pone.0082980	low	low	low	low	low	high
Marlene Santos et al.	10.1089/gtmb.2013.0197	low	low	low	low	higher	low
Yong-Jun Wang et al.	10.1371/journal.pone.0064617	low	low	low	low	low	higher

Mei He et al.	Genetic distribution and association analysis of DRD2 gene polymorphisms with major depressive disorder in the Chinese Han population	low	low	low	low	unclear	low
Yongjun Wang et al.	10.1371/journal.pone.0057042	low	low	low	low	unclear	unclear
Andrea Evinova et al.	10.4149/gpb_2012_049	low	low	low	low	higher	unclear
Suxia Cao et al.	10.3969/j.issn.1673-5374.2012.24.011	low	low	low	low	low	higher
Wenmin Tian et al.	10.1097/YPG.0b013e3283539550	low	low	low	low	low	low
Alessandra Minelli et al.	10.1016/j.jad.2012.01.028	low	low	low	low	low	low
Andrea Vereczkei et al.	10.1016/j.pnpbp.2019.01.006	low	low	low	low	low	low
Shiwan Tao et al.	10.3389/fnins.2018.00827	low	low	low	low	low	low
Jiaxin Hu et al.	10.1097/YPG.0000000000000207	low	low	low	low	high	low
Su-Xia Cao et al.	10.1080/13651501.2017.1406121	low	low	low	low	unclear	high
Duan Zeng et al.	10.2147/NDT.S155076	low	low	low	low	low	unclear
Kyu-Man Han et al.	10.1016/j.pscychresns.2018.01.005	low	low	low	low	unclear	low
Lin Wang et al.	10.1002/ajmg.b.32573	low	low	low	low	low	unclear
Qingzhong Wang et al.	10.1097/YPG.0000000000000185	low	low	low	low	high	low
Lijuan Wang et al.	doi.org/10.1089/gtmb.2016.0426	low	low	low	low	unclear	high
Merve Şahin Can et al.	Psychiatr Danub. 2017 Jun;29(2):179-185.	low	higher	low	low	higher	unclear
Kyu-Man Han et al.	10.1016/j.jpsychires.2017.05.010	low	low	low	low	low	higher
Lachlan Cribb et al.	10.1080/1028415X.2017.1327685	low	low	low	low	low	low
Yanfang Wang et al.	10.1080/87565641.2017.1306527	low	low	low	low	unclear	low
Cheng Xu et al.	10.1016/j.neulet.2017.04.061	low	low	low	low	low	unclear
Dong Han et al.	10.1016/j.jad.2017.04.028	low	low	low	low	low	low
Eunsoo Won et al.	10.1016/j.pnpbp.2017.02.028	low	low	low	low	low	low
Marieke S. Tollenaar et al.	10.1007/s00406-017-0784-z	low	low	low	low	low	low
Jingsong Ma et al.	10.1371/journal.pone.0170994	low	low	low	low	unclear	low
Kyu-Man Han et al.	10.1038/srep42621	low	low	low	low	unclear	unclear
Laura Mandelli et al.	10.1007/s12325-017-0482-2	low	low	low	low	low	unclear
Weibo Niu et al.	10.1097/YPG.0000000000000161	low	low	low	low	unclear	low
EA Bondarenko et al.	Int J Med Sci. 2016 Dec 8;13(12):977-983. eCollection 2016.	low	low	low	low	unclear	unclear
Ninomiya-Baba M et al.	10.1017/neu.2016.66	low	low	low	low	low	unclear
Jian Zhang et al.	10.1016/j.jad.2016.08.083	low	low	low	low	low	low
Rui Zhang et al.	10.1016/j.neulet.2016.10.018	low	low	low	low	unclear	low
Sunyoung Choi et al.	10.1371/journal.pone.0164301	low	low	low	low	unclear	unclear
Jianhua Chen et al.	10.1080/15622975.2016.1245442	low	low	low	low	unclear	unclear
Raheel Mushtaq et al.	10.7759/cureus.673	low	low	low	low	unclear	unclear
Erica L. Tatham et al.	10.1016/j.pscychresns.2016.04.014	low	low	low	low	higher	unclear
Yanfang Wang et al.	Am J Transl Res. 2016 Feb 15;8(2):1281-92. eCollection 2016.	low	low	low	low	low	higher
Milutin Kostic et al.	10.1002/hbm.23165	low	low	low	low	low	low
Zujia Wen et al.	10.1002/ajmg.b.32428	low	low	low	low	unclear	low
Raja Amjad Waheed Khan et al.	10.3109/15622975.2015.1126676	low	low	low	low	unclear	unclear
Zujia Wen et al.	10.1016/j.jad.2016.01.034	low	low	low	low	unclear	unclear

Wenjin Li et al.	10.1016/j.euroneuro.2015.05.004	low	low	low	low	low	unclear
M. Elizabeth Sublette et al.	10.1097/YPG.0000000000000111	low	low	low	low	higher	low
Jianhua Chen et al.	10.1192/bjp.bp.114.151688	low	low	low	low	higher	higher
Ya Bin Wei et al.	10.1016/j.jad.2015.09.025	low	low	low	low	low	higher
Jingsong Ma et al.	10.1016/j.jad.2015.07.041	low	low	low	low	low	low
Jianhua Chen et al.	10.1016/j.jad.2015.06.040	low	low	low	low	unclear	low
Mayuko Onodera et al.	10.1097/YPG.0000000000000096	high	low	low	low	high	unclear
Mei He et al.	Int J Clin Exp Pathol. 2019 Feb 1;12(2):628-639	low	low	low	low	unclear	high
J Peedicayil	10.1038/mp.2014.4	low	higher	low	low	low	unclear
Eva Kitzlerová et al.	10.12659/MSM.907202	low	low	low	low	high	higher
Eunsoo Won June Kang et al.	10.1016/j.psyneuen.2016.07.008	low	low	low	low	low	high
Lina Quteineh et al.	10.1016/j.jad.2016.03.031	low	low	low	low	low	low
Xian-cang Ma et al.	10.1016/j.psychres.2013.08.025	low	low	low	low	unclear	low
Pei-Shen Ho et al.	10.1016/j.pscychresns.2012.04.005	low	low	low	low	higher	unclear
Emmanuel I Sarmiento-Hernández et al.	Actas Esp Psiquiatr. 2019 Jan;47(1):1-6. Epub 2019 Jan 1	low	low	low	low	unclear	higher
Hun Soo Chang et al.	10.30773/pi.2018.11.01	low	low	low	low	unclear	unclear
Sang Min Lee et al.	10.1016/j.gene.2016.12.006	low	low	low	low	high	unclear
Shitao Rao et al.	10.1002/ajmg.b.32423	low	low	low	low	low	high
Honglei Yin et al.	10.1016/j.psychres.2012.11.009	low	low	low	low	low	low
Concetta Crisafulli et al.	10.1007/s11033-012-2220-9	low	low	low	low	low	low
Elzbieta Gałecka et al.	10.1016/j.jpsychires.2012.05.003	low	low	low	low	higher	low
Betina Elfving et al.	10.1097/YPG.0000000000000190	low	low	low	low	higher	higher
Hun Soo Chang et al.	10.1016/j.bbr.2015.06.005	low	low	low	low	low	higher
Maria GabrielaNielsen et al.	10.1016/j.jad.2015.05.003	low	low	low	low	low	low
Ayako Inoue et al.	10.3109/13651501.2015.1043133	low	higher	low	low	higher	low
Meng Wang et al.	10.1016/j.pnpbp.2015.04.001	low	low	low	low	higher	higher
Tanel Traks et al.	10.1002/brb3.314	low	low	low	low	higher	higher
Wenjin Li et al.	10.1002/ajmg.b.32291	low	low	low	low	unclear	higher
Qingzhong Wang	10.4088/JCP.13m08962	low	low	low	low	unclear	unclear
Kenji Hayashi et al.	10.2147/NDT.S61275	low	low	low	low	higher	unclear
David Stacey et al.	10.1016/j.jad.2014.05.001	low	low	low	low	higher	higher
Qingzhong Wang, et al.	10.3109/15622975.2014.915057	low	low	low	low	higher	higher
Thomas W. Frazier et al.	10.1002/brb3.226	low	low	low	low	low	higher
Gul Ozbey et al.	10.1016/j.pharep.2013.09.004	low	low	low	low	higher	low
Aleksandra Szczepankiewicz et al.	10.1016/j.jad.2014.04.002	low	low	low	low	low	higher
Xinhua Shen et al.	10.1016/j.jad.2014.03.008	low	low	low	low	low	low
Le Wang, Cuijuan Shi et al.	10.1016/j.neulet.2014.02.055	low	low	low	low	unclear	low
Wei Jia et al.	10.1371/journal.pone.0087247	low	low	low	low	higher	unclear
Kuanjun He et al.	10.1016/j.pnpbp.2013.12.004	low	low	low	low	low	higher
Daimei Sasayama et al.	10.1016/j.jpsychires.2013.12.002	low	low	low	low	low	low
Xiaoquan Wang etal.	10.1111/pcn.12133	low	low	low	low	higher	low
Chen Zhang et al.	10.1016/j.jad.2013.11.010	low	low	low	low	higher	higher
Hely Kalska et al.	10.1155/2013/849346	low	low	low	low	high	higher

Kuanjun He et al.	10.1192/bjp.bp.113.126979	low	low	low	low	higher	high
David Stacey et al.	10.1097/YPG.0000000000000006	low	low	low	low	higher	higher
Ahdidan J et al.	10.1017/neu.2013.3	low	low	low	low	higher	higher
Birgul Elbozan Cumurcu et al.	10.17305/bjbms.2013.2390	low	low	low	low	higher	higher
Hui Li et al.	10.1016/j.pnpbp.2013.04.012	low	low	low	low	low	higher
Zsuzsa Halmai et al.	10.1016/j.jad.2013.02.033	low	low	low	low	low	low
Zhongchun Liu et al.	10.1038/srep01548	low	low	low	low	low	low
Marina Mitjans et al.	10.1007/s00213-013-2995-y	low	low	low	low	low	low
Weidong Ji et al.	10.1016/j.psychres.2012.09.024	low	low	low	low	higher	low
K Koido et al.	10.1038/tp.2012.74	low	low	low	low	higher	higher
Jun Chen et al.	10.1007/s12035-012-8294-5	low	low	low	low	low	higher
Yingcheng Wang et al.	10.1016/j.psychres.2012.06.024	low	low	low	low	higher	low
Zhenxing Yang et al.	10.1016/j.genhosppsych.2012.05.015	low	low	low	low	higher	higher
Negar Firouzabadi et al.	10.1016/j.psychres.2012.05.002	low	low	low	low	low	higher
A. Carballedo et al.	10.1002/ajmg.b.32060	low	low	low	low	low	low
EK Green et al.	10.1038/mp.2012.48	low	low	low	low	low	low
Tao Li et al.	10.3109/15622975.2011.615860	low	low	low	low	unclear	low
Kinga Bobińska et al.	10.1016/j.jad.2016.03.068	low	low	low	low	higher	unclear
Elzbieta Gałecka et al.	10.1016/j.pharep.2016.04.019	low	low	low	low	higher	higher
HN Buttenschøn et al.	10.1038/tp.2015.167	low	low	low	low	higher	higher
Paulina Wigner et al.	10.1016/j.eurpsy.2018.05.001	low	low	low	low	higher	higher
Paulina Wigner et al.	10.1016/j.eurpsy.2017.10.012	low	low	low	low	unclear	higher
Paulina Wigner et al.	10.1111/jcmm.13459	low	low	low	low	unclear	unclear
Piotr Czarny et al.	10.7717/peerj.5116	low	low	low	low	higher	unclear
Yingyan Hu et al.	10.1016/j.jad.2017.06.059	low	low	low	low	higher	higher
Snezhina Mihailova et al.	10.1016/j.jneuroim.2016.03.005	low	low	low	low	unclear	higher
Patricia Araújo Pereira et al.	10.1002/gps.4018	low	low	low	low	unclear	unclear
Elżbieta Gałecka et al.	10.18388/abp.2015_1002	low	low	low	low	higher	unclear
Piotr Czarny et al.	10.1016/j.jad.2015.05.044	low	low	low	low	unclear	higher
Piotr Gałecki et al.	10.1016/j.pnpbp.2013.04.011	low	low	low	low	higher	unclear
Davide Seripa et al.	10.1016/j.jagp.2012.10.012	low	low	low	low	higher	higher
Amy Froud et al.	10.1080/1028415X.2017.1415281	low	low	low	low	low	higher
Piotr Czarny et al.	10.12659/msm.898091	low	low	low	low	unclear	low
Piotr Gaecki et al.	10.1016/j.jad.2012.10.025	low	low	low	low	unclear	unclear
Warren D. Taylor et al.	10.1002/hbm.21445	low	low	low	low	higher	unclear
Peng Wang et al.	Int J Clin Exp Pathol. 2015 Jan 1;8(1):906-13. eCollection 2015.	low	low	low	low	low	higher
Yong He et al.	10.1089/dna.2012.1660	low	low	low	low	unclear	low
Henrietta Nørmølle et al.	10.1017/neu.2016.26	low	low	low	low	low	unclear
Ana Ching-López et al.	10.2147/NDT.S90369	low	low	low	low	low	low
Fan Yuan et al.	10.1177/0269881114554273	low	low	low	low	low	low
Tao Li et al.	10.3109/15622975.2011.615860	low	low	low	low	higher	Low
