# Supplementary material for: A Systematic Review of Candidate Genes for Major Depression
Source: Medicina (Kaunas). 2022 Feb 14;58(2):285. doi: 10.3390/medicina58020285 (PMC8875554; doi:10.3390/medicina58020285)
Supplement: Supplementary file 1 [file medicina-58-00285-s001.zip › Table S1.pdf]

Table S1. Main characteristics and findings of studies included in the systematic review

No.	Main author	Year of publication	Participants (N) Case/Control	Country	Gene name	rs-id	Chromosome position	p for gene	OR (Cl) for gene	p for allele	OR (Cl) for allele
1	Yang J et al. [79]	2019	278/287	China	TPH2	rs11178997	12q21.1	<0.05		<0.05	
					TPH2	rs120074175	12q21.1	<0.05		<0.05	
					TPH2	rs4570625	12q21.1				
					5-HT2A	rs6311	13q14.2	0,029			1.40(1.09-1.80)
					5-HT2A	rs6313	13q14.2	0,049			
					5-HT2A	rs7997012	13q14.2				1.130 (1.00-0.27)
2	Aldoghachi AF et al. [76]	2019	300/300	Malaysia	BDNF	rs6265	11p14.1	0,008		0.015*	
3	Chao JK et al. [77]	2018	265/223	Taiwan	COMT	rs4633	22q11.21	0,016		0,006	
					COMT	rs4646312	22q11.21	0,001		<0.001	
					COMT	rs4680	22q11.21				1.18 (1.03–1.36)
					COMT	rs737866	22q11.21				
4	Ren D et al. [37]	2017	568/846	China	GRIK4	rs11218016	11q23.3				
					GRIK4	rs4582985	11q23.3				
					GRIK4	rs56275759	11q23.3	0,029	1.37 (1.05-1.8)	0,011	
					GRIK4	rs6589847	11q23.3				
					GRIK4	rs79526501	11q23.3				
5	Liu J et al. [19]	2017	1135/989	China	YWHAE	rs11655548	17p13.3				
					YWHAE	rs12452627	17p13.3				
					YWHAE	rs1532976	17p13.3	0.039*		0.013*	
					YWHAE	rs1873827	17p13.3				
					YWHAE	rs2131431	17p13.3				
					YWHAE	rs28365859	17p13.3				
					YWHAE	rs34041110	17p13.3	0,04		0,036	
					YWHAE	rs3752826	17p13.3				
					YWHAE	rs7224258	17p13.3				
					YWHAE	rs7225165	17p13.3				
					YWHAE	rs8064578	17p13.3				
6	Xie T et al. [80]	2017	437/377	Spain	VEGF	rs10738760	6p21.1				
					VEGF	rs4416670	6p21.1	0,01	1.60 (1.03–2.46)		
					VEGF	rs6921438	6p21.1				0.039 (0.005-0.289)
					VEGF	rs6993770	6p21.1				
7	Bi Y et al. [59]	2017	568/846	China	SLC6A2	rs1362621	16q12.2				
					DRD2	rs2234689	11q23.2				
					SLC6A2	rs2242446	16q12.2				
					SLC6A3	rs2550956	5p15.33	0,04		0,033	
					SLC6A3	rs3863145	5p15.33				
					SLC6A2	rs5564	16q12.2				
					DRD2	rs7131056	11q23.2				
8	Dadkhah T et al. [38]	2016	250/250	Iran	GRM4	rs2229901	6p21.31	0,018	1.46((1.12-1.91)	0,007	
9	Mahmood S et al. [81]	2016	111/207	Slovakia	TP53	rs1042522	17p13.1				3.459 (1.909 - 6.266)
					IGFBP3	rs285474	7p12.3				
					EGF	rs4444903	4q25				

10	Pérez-Olmos I et al. [22]	2016	87/131	Colombia	SLC6A4	5-HTT polymorphism	17q11.2			0.038*	
11	Treutlein J et al. [42]	2017	595/1295	Germany	NPY2R	rs6857715	4q32.1			0,02	
12	Sarubin N et al. [17]	2016	412/634	Germany	NR3C1	rs10052957	5q31.3				
					CRHR1	rs110402	17q21.31				
					FKBP5	rs1360780	6p21.31				
					NR3C2	rs2070951	4q31.23				
					CRHR1	rs242924	17q21.31				
					CRHR1	rs242939	17q21.31				
					NR3C1	rs41423247	5q31.3				
					FKBP5	rs4713916	6p21.31				
					CRHR1	rs4792887	17q21.31				
					NR3C2	rs5522	4q31.23				0,5567
					NR3C1	rs6189	5q31.3				
					NR3C1	rs6190	5q31.3				
					NR3C1	rs6195	5q31.3	0.002*			
					NR3C1	rs6198	5q31.3				
					CRHR1	rs7209436	17q21.31				
13	Zhang C et al. [82]	2016	772/759	China	IL6	rs1524107	7p15.3				
					IL6	rs1800795	7p15.3				
					IL6	rs1800796	7p15.3				
					IL6	rs1800797	7p15.3	0,002	4.76 (1.61–14.07	0,002	
					IL6	rs2069837	7p15.3				
14	Sayadi MA et al. [83]	2016	208/187	Tunisia	MTHFR	rs1801133 (C677T)	1p36.22	0,047	0.655(0.432–0.995)		
15	Sun N et al. [46]	2016	459/412	China	SLC6A4	5-HTTLPR	17q11.2	0.04			
					BDNF	rs6265	11p14.1				
16	Zhang Z et al. [86]	2015	744/767	China	NDUFV2	rs12457810	18p11.22				
					NDUFV2	rs12964485	18p11.22	0,012			
					NDUFV2	rs2279992	18p11.22				
					NDUFV2	rs2377961	18p11.22				
					NDUFV2	rs4797356	18p11.22	0,02			0.785 (0.695 – 0.886)
					NDUFV2	rs4798765	18p11.22	0,013			1.386 (1.186 – 1.622)
					NDUFV2	rs4798772	18p11.22				0.696 (0.579 – 0.835)
					NDUFV2	rs8084822	18p11.22				
					NDUFV2	rs874250	18p11.22				
17	Zhang L et al. [84]	2015	292/208	China	ABCB1	rs1045642	7q21.12				
					ABCB1	rs1128503	7q21.12				
					ABCB1	rs2032582	7q21.12			0,03	
					ABCB1	rs2032583	7q21.12			0,037	
					ABCB1	rs2235015	7q21.12				
					ABCB1	rs2235040	7q21.12				
18	Wei Y et al. [169]	2015	456/582	China	CREB1	rs16839883	2q33.3	<0.001		0,005	
					CREB1	rs2254137	2q33.3				
19	Wang Y et al. [60]	2015	388/388	China	NET (SLC6A2)	rs2242446 (T-182C)	16q12.2				1.678 (1.264–2.226)
					NET (SLC6A2	rs5569 (G1287A)	16q12.2				
					MIRLET 7I	rs10877887	12q14.1	0,03			

20	Liang Y et al. [85]	2015	237/296	China	the promoter region	rs13293512	12q14.1	0,015		0,03	
21	Du T et al. [23]	2015	738/1098	China	METTL3	rs1139130	14q11.2				
					METTL14	rs11945703	4q26				
					WTAP	rs12204454	6q25.3				
					METTL3	rs1263790	14q11.2				0.77 (0.65–0.92)
					METTL3	rs1263791	14q11.2				
					ALKBH5	rs12936694	17p11.2	0,0022		0.0008	
					WTAP	rs1440	6q25.3				1.385 (1.062–1.806
					METTL14	rs17197156	4q26	0,0315		0,0156	
					ALKBH5	rs2124370	17p11.2				
					METTL3	rs2242526	14q11.2				
					WTAP	rs2758313	6q25.3				
					METTL14	rs298977	4q26				
					METTL14	rs298996	4q26				
					METTL3	rs3752411	14q11.2				
					METTL14	rs441216	4q26				
					METTL3	rs4417466	14q11.2				
					ALKBH5	rs4924839	17p11.2				
					ALKBH5	rs4925144	17p11.2				
					ALKBH5	rs8068517	17p11.2				
					ALKBH5	rs9913266	17p11.2				
					FTO	rs9939609	16q12.2				
22	Kokut S et al. [89]	2015	144/105	Turkey	ADRB1	rs1801252	10q25.3				
					ADRB1	rs1801253	10q25.3				2.75 (0.876-8.637)
23	Watanabe S et al. [1]	2015	216/213	Japan	SLC6A4	5-HTTLPR	17q11.2				
24	Congiu C et al. [87]	2014	590/441	Italy	KCNK2	rs10494996	1q41				
					KCNK2	rs10779646	1q41	0,002	1.42 (1.10–1.85)	0,01	
					KCNK2	rs12136349	1q41				
					KCNK2	rs2841608	1q41				
					KCNK2	rs2841616	1q41				
					KCNK2	rs7549184	1q41				
25	Zhou Y et al. [88]	2014	313/318	China	DBH	DBH5′-Ins/Del	9q34.2	0,007			
26	Hua P et al. [92]	2014	105/485	China	CRY2	rs10838524	11p11.2				
					CRY1	rs2287161	12q23.3	0,01		0,012	
					TEF	rs738499	22q13.2	<0.001		0,001	
27	McFarquhar M et al. [90]	2014	649/571	United Kingdom	TOMM40	rs2075650	19q13.32				1.366 (1.083–1.723)
28	Nazree NE et al. [24]	2013	265/332	Malaysia	TPH2	rs13864494	12q21.1				1.638 (1.294–2.073)
					TPH2	rs1386495	12q21.1				0.124 (0.094–0.164)
					TPH2	rs7305115	12q21.1				
29	Mocking R et al. [91]	2013	137/73	The Netherlands	FABP2	rs1799883	4q26				

30	Santos M et al. [20]	2014	80/160	Portugal	ABCB1	rs1045642 (C3435T)	7q21.12				1.598 (1.212–2.107)
					ABCB1	rs1128503 (C1236T)	7q21.12				
					ABCB1	rs2032582 (G2677TA)	7q21.12	0.049*			
31	Wang Y et al. [95]	2013	700/673	China	GAL	rs1042577	11q13.2				
					GAL	rs1546309	11q13.2				
					GAL	rs2097042	11q13.2				
					GAL	rs2187331	11q13.2				1,524
					GAL	rs2510387	11q13.2				
					GAL	rs2513297	11q13.2				
					GAL	rs3136540	11q13.2				
					GAL	rs4432027	11q13.2				
					GAL	rs694066	11q13.2	0,0000673		0,000097	
					GAL	rs948854	11q13.2				
32	He M et al. [93]	2013	114/224	China	DRD2	rs1799732	11q23.2				
					DRD2	rs1800497	11q23.2				
					DRD2	rs6277	11q23.2				2.06 (1.09–3.92)
33	Evinova A et al. [94]	2012	134/143	Slovakia	MTHFR	rs1801133 - rs1801131 (A1298C)	1p36.22				
					BDNF	rs6265	11p14.1	0,032	2.38 (1.07 - 5.32)	0,019	
34	Cao S et al. [100]	2012	315/278	China	ADCY9	rs2230739	16p13.3				
35	Tian W et al. [96]	2012	463/413	China	EGF	rs11568943	4q25				
					EGF	rs11569017	4q25	0,0039	2.09 (1.05–4.16)		
					EGF	rs11569126	4q25				
					EGF	rs2237043	4q25				
					EGF	rs2237051	4q25				
					EGF	rs2250724	4q25				
					EGF	rs4444903	4q25				
36	Minelli A et al. [97]	2012	522/375	Italy	PCLO	rs2522833	7q21.11	0,016			
37	Vereczkei A et al. [98]	2019	195/406	Hungary	P2RX7	rs1653625	12q24.31				
					P2RX7	rs1718106	12q24.31				
					P2RX7	rs1718119	12q24.31				
					P2RX7	rs2230911	12q24.31				
					P2RX7	rs2230912	12q24.31				
					P2RX7	rs3751143	12q24.31				
38	Tao S et al. [99]	2018	185/64	China	TPH2	rs11178998	12q21.1				
					TPH2	rs17110747	12q21.1				
					TPH2	rs41317118	12q21.1				
					TPH2	rs4290270	12q21.1	0.0167			
					TPH2	rs4570625	12q21.1				
					TPH2	rs7305115	12q21.1				
39	Cao SX et al. [61]	2018	504/303	China	NET (SLC6A2	rs2242446	16q12.2				
					NET (SLC6A2	rs5569	16q12.2				
40	Zeng D et al. [101]	2018	225/436	China	RGS4	rs10759	1q23.3				
					MIR124-1	rs531564	8p23.1				
					RGS4	rs951436	1q23.3				
41	Han KM et al.	2018	95/65	Republic of	SLC6A4	5- HTTLPR	17q11.2				

41	[55]	2018	[illegible]	Korea	BDNF	rs6265	11p14.1				
42	Wang L et al. [25]	2017	1056/1248	China	NDST3	rs10857057	4q26				
					NDST3	rs11098403	4q26				
					NDST3	rs2389521	4q26				
					NDST3	rs3817274	4q26				
					NDST3	rs4833564	4q26				
					NDST3	rs609512	4q26				
					NDST3	rs6837896	4q26				
					NDST3	rs687492	4q26				
					NDST3	rs7689157	4q26				
43	Wang Q et al. [105]	2017	1045/1235	China	ZNF804A	rs10497655	2q32.1				
					ZNF804A	rs12476147	2q32.1				
					ZNF804A	rs12477430	2q32.1				
					ZNF804A	rs13393273	2q32.1				
					ZNF804A	rs1344706	2q32.1				
					ZNF804A	rs1366842	2q32.1				
					ZNF804A	rs17508595	2q32.1				
					ZNF804A	rs17617913	2q32.1	0,045		0,024	
					ZNF804A	rs2054548	2q32.1				
					ZNF804A	rs2170203	2q32.1				
					ZNF804A	rs2369593	2q32.1				
					ZNF804A	rs4667001	2q32.1				
					ZNF804A	rs7597593	2q32.1				
44	Wang L et al. [106]	2017	67/44	China	SLC6A15	rs1545843	12q21.31				
45	Can MŞ et al. [102]	2017	86/89	Turkey	VDR	rs2228570	12q13.11				1,502
46	Han KM et al. [103]	2017	105/85	Republic of Korea	TESC	rs7294919	12q24.22				
47	Cribb L et al. [104]	2017	154/53	Australia	FADS1	rs174537	11q12.2	<0.05	0.575 (.338, 0.977)		
					FADS1	rs174547	11q12.2	<0.05	0.584 (.343, 0.994)		
					FADS2	rs174570	11q12.2				
					FADS2	rs174575	11q12.2				
					FADS2	rs3984458	11q12.2				
					FADS2	rs498793	11q12.2				
48	Wang Y et al. [112]	2017	75/45	China	MAPK1	rs12678428	22q11.22				
					PTPRR	rs1513105	12q15				
49	Xu C et al. [113]	2017	381/291	China	MIR34C-MIR34B	rs11606481	11q23.1				
					MIR34C-MIR34B	rs2187473	11q23.1				1,526
					MIR34C-MIR34B	rs28757623	11q23.1				
					MIR34C-MIR34B	rs4938723	11q23.1				
50	Han D et al. [114]	2017	274/273	China	VEGF	rs2010963	6p21.1	0,03			
51	Won E et al. [107]	2017	103/83	Republic of Korea	VMAT1	rs1390938	8p21.3				
52	Tollenaar MS et al. [107]	2017	428/387	The Netherlands	OXTR	rs2254298	3p25.3				
					OXTR	rs2268498	3p25.3				
					OXTR	rs53576	3p25.3				
					GNB3	rs5443	12p13.31	<0.00		<0.00	
53	Ma J et al.	2017	512/513	China	CREB1	rs11904814	2q33.3				
					CREB1	rs2253206	2q33.3				

[illegible]	[109]	[illegible]	[illegible]	[illegible]	CREB1	rs2551941	2q33.3			
					CREB1	rs6740584	2q33.3			
54	Han KM et al. [110]	2017	114/88	Republic of Korea	FKBP5	rs1360780	6p21.31			
55	Mandelli L et al. [111]	2017	238/324	Republic of Korea	SIGMAR 1	rs1800866	9p13.3			
56	Bondarenko EA et al. [117]	2016	150/200	Russia	ACE	rs4646994 (In/del)	17q23.3			
					MTHFR	rs1801133	1p36.22			
					GNB3	rs5443	12p13.31			1,898
					BDNF	rs6264	11p14.1			
57	Ninomiya-Baba M et al. [26]	2016	799/1189	Japan	KLF9	rs11142387				
					GP2	rs12597579			0,042	0.789 (0.673–0.924)
					TMEM	rs6548238		0,033		
58	Zhang J et al. [21]	2016	191/2000	China	ESR2	rs1256049	14q23.2-q23.3	0.004*	0.005*	
					ESR2	rs4986948	14q23.2-q23.3			
59	Choi S et al. [115]	2016	86/64	Republic of Korea	SLC6A15	rs1545843	12q21.31			
60	Mushtaq R et al. [116]	2016	240/160	India	TPH1	rs1799913 (A779C)	11p15.1		≤ 0.0001	
61	Tatham EL et al. [56]	2016	55/18	Canada	SLC6A4	5-HTTLPR	17q11.2			
					BDNF	rs4680	11p14.1			
62	Wang Y et al. [47]	2016	200/199	China	SLC6A4	5-HTT LPR	17q11.2	0,034	0,009	
					SLC6A4	5-HTT VNTR	17q11.2			
63	Kostic M et al. [48]	2016	77/66	Serbia	SLC6A4 (5-	rs25531	17q11.2	0,04		
					COMT	rs4680	22q11.21	0.04		
					BDNF	rs6265	11p14.1	0,001		
64	Wen Z et al. [121]	2016	1056/1248	China	NRG1	rs11989919	8p12			
					NRG1	rs12680129	8p12			
					NRG1	rs243928	8p12			
					NRG1	rs2466050	8p12			
					NRG1	rs2466064	8p12			
					NRG1	rs2919375	8p12	2,50E-04		
					NRG1	rs2954042	8p12			
					NRG1	rs3924999	8p12		0,048	
					NRG1	rs4236710	8p12			
					NRG1	rs4445183	8p12			
					NRG1	rs4512342	8p12	0,036		1.2610(1.045-1.522)
					NRG1	rs4531002	8p12			
					NRG1	rs4733365	8p12			
					NRG1	rs6982890	8p12			
					NRG1	rs7829383	8p12	0,045		0.834 (0.74-0.94)
65	Khan RAW et al. [27]	2016	1056/1248	China	SLCO6A 1	rs2400795	5q21.1			1.81 (1.40–2.34)
					SLCO6A 1	rs6878284	5q21.1			
					SLCO6A 1	rs7734060	5q21.1	0,011	0,04	
					SLCO6A 1	rs981988	5q21.1			
66	Wen Z et al. [122]	2016	1056/1248	China	NRGN	rs12278912	11q24.2	133	0,0034	
					NRGN	rs12807809	11q24.2	0,0381	0,0367	
					GRM8	rs10487457	7q31.33			

67	Li W et al. [15]	2015	1045/1235	China	GRM8	rs10487459	7q31.33			
					GRM7	rs11928865	3p26.1			
					GRM8	rs1361995	7q31.33		1,13E-04	
					GRM7	rs17031835	3p26.1			
					GRM7	rs2229902	3p26.1			
					GRM8	rs2237748	7q31.33			
					GRM8	rs2237781	7q31.33			
					GRM8	rs2237797	7q31.33			
					GRM8	rs2299472	7q31.33			
					GRM7	rs3749380	3p26.1			
					GRM7	rs3792452	3p26.1			
					GRM7	rs779701	3p26.1			
					GRM7	rs779706	3p26.1		7,16E-04	
					GRM7	rs9870680	3p26.1			
68	Sublette ME et al. [118]	2016	635/480	USA, Spain	FADS1	rs174537	11q12.2			
					FADS1	rs174545	11q12.2			
					FADS1	rs174546	11q12.2			
					FADS1	rs174547	11q12.2			
					FADS1	rs174556	11q12.2			
					FADS1	rs174561	11q12.2			
					FADS2	rs174568	11q12.2			
					FADS2	rs174570	11q12.2			
					FADS2	rs174583	11q12.2			
					FADS2	rs174589	11q12.2			1.512 (1.133–2.017)
					FADS2	rs174602	11q12.2			1.329 (1.127–1.567)
					FADS2	rs174620	11q12.2			
					FADS2-FADS3	rs174627	11q12.2			
					FADS2	rs2072114	11q12.2			
					FADS2	rs2524299	11q12.2			
					FADS2	rs526126	11q12.2			1.14 (1.005–1.292)
					FADS2	rs968567	11q12.2			
					FADS2	rs99780	11q12.2			0,657
69	Wei YB et al. [119]	2015	436/1590	Sweden	hTERT	rs2736100	5p15.33			
70	Ma J et al. [120]	2015	289/289	China	TPH2	rs11178997	12q21.1	<0.05	<0.05	
					TPH2	rs120074175	12q21.1	<0.05	<0.05	
					TPH2	rs4570625	12q21.1			
71	He M et al. [126]	2019	334/497	China	DRD2	rs1076560	11q23.2			
					DRD2	rs10891556	11q23.2			
					DRD2	rs12574471	11q23.2			0.869 (0.761–0.991)
					DRD2	rs1799978	11q23.2			
					DRD2	rs1800479	11q23.2			
					DRD2	rs2075652	11q23.2			1.295 (1.029–1.630)
					DRD2	rs2242591	11q23.2			
					DRD2	rs2734833	11q23.2			

					DRD2	rs4436578	11q23.2			
					DRD2	rs4460839	11q23.2			
					DRD2	rs4586205	11q23.2			
					DRD2	rs4648317	11q23.2		0,04	
					DRD2	rs4648318	11q23.2			
					DRD2	rs4648319	11q23.2			
					DRD2	rs6279	11q23.2			2.14 (1.12–4.08)
					DRD2	rs7131056	11q23.2		0,02	
72	Milaneschi Y et al. [127]	2014	1544/336+2470	Netherlands	FTO	rs9939609	16q12.2	0,023		
73	Kitzlerová E et al. [50]	2018	68/90	Czech Republic	HSPA1A	rs1008438	6p21.3		0,026	
					HSPA1A	rs1043618	6p21.3		0,038	
					SLC6A4	rs4795541	17q11.2			
					SLC6A4	rs57098334	17q11.2			1,261
					BDNF	rs6265	11p14.1			
					HTR2A	rs6311	13q14-q21			
					APOE	rs7412-rs429358	19q13.31			
74	Won E et al. [123]	2016	52/52	Republic of Korea	NR3C1	rs41423247	5q31.3			
75	Quteineh L et al. [124]	2016	CoLaus|PsyCoLaus rs6510997 - 1431/1916<br>The radiant study rs3746266 - 2142/700; rs2075017 - 1352/ 808<br>NESDA/NTR study rs3746266 - 1768/2895 rs6510997 - 1768/ 2895	Switzerland	CRTC1	rs2075017	19p13.11			
					CRTC1	rs3746266	19p13.11			
					CRTC1	rs6510997	19p13.11			
					CRTC1	rs7257846T	19p13.11			
76	Ma XC et al. [28]	2013	218/514	China	GDNF	rs2216710	5p13.2			1,487
					GDNF	rs2910702	5p13.2			
					GDNF	rs2910709	5p13.2			2,191
					GDNF	rs2973050	5p13.2			
					GDNF	rs3812047	5p13.2			
					GDNF	rs884344	5p13.2			
77	Ho PS et al. [49]	2012	40/12	Taiwan	SLC6A4	rs25531	17q11.2			
					SLC6A4	rs6354	17q11.2			
78	Sarmiento-Hernández EI et al. [53]	2019	200/235	Mexico	SLC6A4	5-HTTLPR polymorphism	17q11.2	0,004	0,009	2.05 (1.48–3.65)
79	Chang HS et al. [125]	2015	270/204	Republic of Korea	ARRB1	rs12274033	11q13.4			
					ARRB1	rs2279130	11q13.4			
					ARRB1	rs34867163	11q13.4			
					ARRB1	rs35382855	11q13.4			
					ARRB1	rs480174	11q13.4			
					ARRB1	rs647630	11q13.4			
					ARRB1	rs877711	11q13.4			1.152 1.001–1.325
80	Lee SM et al.	2018	13/20	Republic of	TPH1	rs1799913	11p15.1			

[illegible]	[133]	[illegible]	[illegible]	Korea	TPH1	rs1800532	11p15.1			
81	Rao S et al. [51]	2016	36/141	Hong Kong	TEF	rs2234058	22q13.2			
					TEF	rs2234059	22q13.2			
					TEF	rs5758321	22q13.2			
					HOMER 1	rs60029191	5q14.1			
					SLC6A4	rs6354	17q11.2		0,022	
					HOMER 1	rs6865475	5q14.1			4.72 (1.60–13.89)
					TEF	rs738499	22q13.2			
82	Yin H et al. [39]	2016	219/57	USA	SLC6A1	rs1062246	3p25.3	0.02		
					GABBR2	rs10739615	9q22.33	0,04		
					SLC1A2	rs10836356	11p13			
					GABRA4	rs12506608	4p12			
					GLS	rs13035504	2q32.2			
					GABRD	rs13303016	1p36.33	0,04		
					GABRA5	rs140685	15q12			5.496 (3.764–8.027)
					GRIN2A	rs1420040	16p13.2	0,02		
					SLC1A3	rs1529461	5p13.2			
					GAD2	rs2236418	10p12.1			
					GABBR2	rs2808536	9q22.33			
					GRIK1	rs363527	21q21.3	0,03		
					GRIN2A	rs3785185	16p13.2	0,01		
					GABRG2	rs424740	5q34			
					GRIN2A	rs4780886	16p13.2			
					GABRA4	rs6447520	4p12			
					GRIA2	rs6849345	4q32.1	0,04		
					GABRB3	rs8041610	15q12			1.136 (1.00-1.29)
					GABRG1	rs976156	4p12			
83	Crisafulli C et al. [29]	2012	145/170	Republic of Korea	ABCB1	Not reported	7q21.12			
					ABCB4		7q21.12			
					TAP2		6p21.32 4q12			
					CLOCK		4p16.3			
					CPLX1		5q35.2			
					CPLX2		3p25.2 8p12			
					SYN2		5q12.3			
					NRG1		10q11.22			
84	Gałecka E et al. [71]	2012	181/149	Poland	NR3C1	rs41423247 (BclI)	5q31.3	0,00001	< 0.00001	0.67 (0.42-1.06)
					NR3C1	rs6189 and rs6190 (ER22/23EK)	5q31.3	0,042	0,01	
					NR3C1	rs6195 (N363S)	5q31.3	0,023	0,005	
85	Elfving B et al. [134]	2012	162/289	Denmark	BDNF	rs11030107	11p14.1			
					BDNF	rs11030109	11p14.1			
					BDNF	rs4923468	11p14.1			
					BDNF	rs6265	11p14.1			
					BDNF	rs7103873	11p14.1			
					BDNF	rs908867	11p14.1			
86	Chang HS et al. [128]	2015	149/193	Republic of Korea	CRH	rs3832590	8q13.1			
					CRH	rs6159	8q13.1			
					CRH	rs77032924	8q13.1			

87	Nielsen MG et al. [129]	2015	414/259	Italy	COMT	rs1801131-rs1801133 (A1298C)	22q11.21	0,03		
					MTHFR	rs1801133(C677T)	1p36.22			
88	Inoue A et al. [130]	2015	280/398	Japan	TMEM132D	rs10847832	12q24.33			
					TMEM132D	rs11060369	12q24.33			
					GABRA6	rs3219151	5q34	1.75E – 04		2.15E – 05
					COMT	rs4680	22q11.21	0,03		
					TMEM132D	rs7309727	12q24.33			
89	Traks T et al. [131]	2015	391/389	Estonia	IKBKE	rs11117909	1q32.1			
					IKBKE	rs11118092	1q32.1			
					IKBKE	rs11118132	1q32.1			
					IKBKE	rs1539243	1q32.1			
					IKBKE	rs15672	1q32.1			
					IKBKE	rs17020312	1q32.1			
					IKBKE	rs17021877	1q32.1			
					IKBKE	rs1930437	1q32.1			<0.001
					IKBKE	rs1930438	1q32.1			
					IKBKE	rs1953090	1q32.1			
					IKBKE	rs2274902	1q32.1			0,001
					IKBKE	rs2297543	1q32.1			
					IKBKE	rs2336940	1q32.1			
					IKBKE	rs3748022	1q32.1			
90	Li W et al. [132]	2015	1045/1235	China	ACSM1	rs163234	16p12.3	1,36E-03		7,22E-04
					ACSM1	rs2301672	16p12.3			
					ACSM1	rs234993	16p12.3			
					ACSM1	rs3087812	16p12.3			
					ACSM1	rs433598	16p12.3			
					ACSM1	rs9930187	16p12.3			
91	Wang Q et al. [76]	2015	1045/1520	China	SNAP25	rs3746544	20p12.2	0,0145	1.213 (1.054-	0,00684
					SNAP25	rs3787283	20p12.2	0,002764	0.8017 (0.71-0.91)	0,000553
92	Hayashi K et al. [171[	2014	30/30	Japan	COMT	rs4680	22q11.21			
					BDNF	rs6265	11p14.1			
93	Stacey D et al. [139]	2014	171/512	Germany	DISC1	rs28930675	1q42.2			
					DISC1	rs3738401	1q42.2			
					DISC1	rs6675281	1q42.2			
					DISC1	rs821616	1q42.2			
94	Wang Q et al. [30]	2014	1045/1235	China	CMYA	rs1129770	5q14.1			
					CMYA	rs12514461	5q14.1	0,000301		9,08E-05
					CMYA	rs1643992	5q14.1			
					CMYA	rs16877062	5q14.1			
					CMYA	rs16877109	5q14.1			
					CMYA	rs16877187	5q14.1			
					CMYA	rs2115127	5q14.1			
					CMYA	rs259127	5q14.1	0,000101		0,000097
					CMYA	rs3828611	5q14.1			
					CMYA	rs4371733	5q14.1			
					CMYA	rs4704591	5q14.1			
					CMYA	rs6453482	5q14.1			
					CMYA	rs6870619	5q14.1			

					CMYA	rs6883197	5q14.1				
					CMYA	rs7343	5q14.1	0,000153		4,15E-05	
95	Frazier TW et al. [140]	2014	48/149	USA	CACNA1C	rs1006737	12p13.33				
					ANK3	rs10994336	10q21.2				2.216 (1.472–3.337)
					DGKH	rs1170191	13q14.11				
					BDNF	rs6265	11p14.1				
96	Ozbey G et al. [141]	2014	54/70	Turkey	ABCB1	rs1045642 (3435C/T)	7q21.12	0,006		0,02	1.444 (1.095–1.904)
97	Szczepankiewicz A et al. [142]	2014	218/742	Poland	FKBP5	rs1360780	6p21.31	0,011			
					NR3C1	rs3800373	5q31.3				
					FKBP5	rs4713916	6p21.31	0,038			
					FKBP5	rs755658	6p21.31				
					FKBP5	rs7748266	6p21.31				1.488 (1.215–1.823)
					FKBP5	rs9296158	6p21.31	0,03			
					FKBP5	rs9394309	6p21.31	0,018			
					FKBP5	rs9470080	6p21.31	0,007			
98	Shen X et al. [143]	2014	368/219	China	MTHFR	rs1801133	1p36.22	<0.001	3.66 (2.53–5.28)	< 0.001	
					COMT	rs4680	22q11.21	0.02	1.52 (1.04–2.21)		
99	Wang L et al. [18]	2014	517/455	China	EHD3	rs1540926	2p23.1				
					EHD3	rs2295471	2p23.1				
					EHD3	rs3769621	2p23.1				0.861 (0.757–0.980)
					EHD3	rs590557	2p23.1				
					EHD3	rs597800	2p23.1				
					EHD3	rs619002	2p23.1			0.0045*	
					EHD3	rs644926	2p23.1			0.0074*	
					EHD3	rs649729	2p23.1				
					EHD3	rs669195	2p23.1				
					EHD3	rs672364	2p23.1				
					EHD3	rs6734084	2p23.1				
100	Jia W et al. [135]	2014	409/619	China	GRM3	rs13242038	7q21.11-q21.12	0,011			7.909 (5.987-10.447)
					GRM3	rs1468412	7q21.11-q21.12				
					GRM3	rs1716070	7q21.11-q21.12				
					GRM3	rs1989796	7q21.11-q21.12				
					GRM3	rs1990040	7q21.11-q21.12				
					GRM3	rs2189814	7q21.11-q21.12				1,54
					GRM3	rs2237558	7q21.11-q21.12				
					GRM3	rs2237562	7q21.11-q21.12				
					GRM3	rs2282966	7q21.11-q21.12				
					GRM3	rs2299225	7q21.11-q21.12				
					GRM3	rs274618	7q21.11-q21.12				
					GRM3	rs274622	7q21.11-q21.12				

					GRM3	rs6465088	7q21.11-q21.12			
					GRM3	rs6947784	7q21.11-q21.12			
					GRM3	rs724226	7q21.11-q21.12			
					GRM3	rs7789655	7q21.11-q21.12			
					GRM3	rs917071	7q21.11-q21.12			
101	Sasayama D et al. [136]	2013	39/40	Japan	ITIH3	rs2535629	3p21.1			
102	Wang X et al. [137]	2013	97/103	China	MTHFR	rs1801133	1p36.22			
					COMT	rs4680	22q11.21			
103	Zhang C et al. [138]	2013	790/725	China	BCL2	rs1564483	18q21.33			
					BCL2	rs1801018	18q21.33			
					BCL2	rs2279115	18q21.33			
104	Kalska H et al. [57]	2013	19/19	Finland	SLC6A4	5-HTTLPR	17q11.2			
105	Stacey D et al. [52]	2013	304/312	Germany	SLC6A4 (5-	rs25531	17q11.2	0.019		
106	Ahdidan J et al. [58]	2013	23/33	Denmark	SLC6A4 (5-	rs25531	17q11.2			
107	Cumurcu BE et al. [144]	2013	80/96	Turkey	SOD2	Ala-Val MnSOD	6q25.3			
					GPX1	rs1050450 (Pro 197 Leu GSH-Px)	3p21.31		0,045	
108	Li H et al. [16]	2013	574/642	China	DCNP1	rs10061623	5q31.1			
					DCNP1	rs12518053	5q31.1			
					DCNP1	rs12520799	5q31.1			
					DCNP1	rs12520809	5q31.1			
					DCNP1	rs13154143	5q31.1			1.637 (1.290-2.078)
					DCNP1	rs17168357	5q31.1			1.983(1.312–2.997)
					DCNP1	rs17168361	5q31.1			
					DCNP1	rs4976298	5q31.1			
109	Halmai H et al. [145]	2013	195/373	Hungary		rs1653625	12q24.31			
					P2RX7	rs2230912	12q24.31			
110	Mitjans M et al. [146]	2013	319/150	Spain	CNR1	rs1049353	6q15			
					CNR1	rs1535255	6q15			
					CNR1	rs806368	6q15			0.881 (0.78-1.00)
					CNR1	rs806371	6q15			1.148 (1.02-1.29)
					CNR1	rs806377	6q15			
111	Ji W et al. [31]	2012	1135/1135	China	CNTNAP2	rs10246256	7q35-q36.1			
					CNTNAP2	rs10952659	7q35-q36.1			
					CNTNAP2	rs17236239	7q35-q36.1			
					CNTNAP2	rs1922892	7q35-q36.1	0,0121	0,00399	
					CNTNAP2	rs2538976	7q35-q36.1			
					CNTNAP2	rs2538991	7q35-q36.1		0,0229	
					CNTNAP2	rs2710102	7q35-q36.1	0,0108	0,00308	
					CNTNAP2	rs2710117	7q35-q36.1	0,00973	0,00309	
					CNTNAP2	rs4431523	7q35-q36.1			1.4 (1.1–1.9)
					CNTNAP2	rs6464774	7q35-q36.1		0,0437	
					CNTNAP2	rs759178	7q35-q36.1	0,0112	0,00631	
					CNTNAP2	rs7794745	7q35-q36.1			

					CNTNAP2	rs851715	7q35-q36.1			0,045	
					CNTNAP2	rs986062	7q35-q36.1			0,0441	
112	Chen J et al. [147]	2012	488/480	China	DAOA	rs1421292	13q33.2; 13q34				
					DAOA	rs2391191	13q33.2; 13q34			0,01	
					DAOA	rs3916965	13q33.2; 13q34				
					DAOA	rs3916966	13q33.2; 13q34				
					DAOA	rs3916967	13q33.2; 13q34				
					DAOA	rs3918341	13q33.2; 13q34			0,04	
					DAOA	rs3918342	13q33.2; 13q34				
					DAOA	rs778294	13q33.2; 13q34			0,02	
					DAOA	rs947267	13q33.2; 13q34				
					DAOA	rs9558562	13q33.2; 13q34				
113	Wang Y et al. [33]	2012	403/475	China	DRD2	rs1076562	11q23.2	<0.001		0,003	
					DRD2	rs1079595	11q23.2				
					DRD2	rs11214607	11q23.2	0,01		0,004	
					DRD2	rs1799978	11q23.2				
					DRD2	rs2075652	11q23.2	0,01			
					DRD2	rs2440390	11q23.2	0,001			
					DRD2	rs2734833	11q23.2	<0.001		<0.001	
					DRD2	rs4460839	11q23.2	0,05		0,02	
					DRD2	rs7122454	11q23.2				
114	Yang Z et al. [6]	2012	397/473	China	REEP5	rs149192	5q22.2				
					REEP5	rs153549	5q22.2				
					APC	rs2229992	5q22.2				
					APC	rs2439591					
					APC	rs2464805	5q22.2			7,07E-07	
					APC	rs42427	5q22.2				
					SRP19	rs495794				0,02013	
					APC	rs563556	5q22.2			1,19E-05	
					REEP5	rs6864403	5q22.2				
					REEP5	rs818427				0,02539	
115	Firouzabadi N et al. [148]	2012	191/104	Iran	ACE	C3123A	17q23.3	0,022			1.52 (1.07 – 2.17)
					ACE	rs4291 (A-240T)	17q23.3	0,026			1.237 (1.093–1.399)
					ACE	rs4343 (A2350G)	17q23.3	0,048		0,015	
116	Carballedo A et al. [149]	2012	37/42	Ireland	BDNF	rs6265	11p14.1				
117	Green EK et al. [150]	2012	1159/2592	United Kingdom	SYNE1	rs9371601	6q25.2				
118	Bobińska K et al. [158]	2016	203/99	Poland	MMP7	rs11568818 (A-181G)	11q22.2				
					MMP2	rs2285053 (C-735T)	16q12.2				
					MMP9	rs3918242 (C1562T)	20q13.12			0,008	
					TIMP2	rs8179090 (G-418C)	17q25.3	0,044	0,707	0,049	
					MMP9	T-1702A	20q13.12	0.001	3,012	<0.001	

119	Gałecka E et al. [159]	2016	139/69	Poland	DIO1	rs11206244	1p32.3				
					DIO1	rs12095080	1p32.3				
					DIO3	rs17716499	14q32.31				
					DIO3	rs7159269	14q32.31				
120	Buttenschøn HN et al. [69]	2015	25/46	Denmark	SORT1	rs11581665	1p13.3; 1p21.3-p13.1	0,005	0.09 (0.16; − 0.03)		
					SORT1	rs12037569	1p13.3; 1p21.3-p13.1				
					SORT1	rs17585355	1p13.3; 1p21.3-p13.1				
					SORT1	rs17646665	1p13.3; 1p21.3-p13.1				
					SORT1	rs3768497	1p13.3; 1p21.3-p13.1	0,027	0.06 (0.12; − 0.007)		
					MYBPHL	rs413582	1p13.3				
					SORT1	rs464218	1p13.3; 1p21.3-p13.1				
					BDNF	rs6265	11p14.1				
					SORT1	rs7536292	1p13.3; 1p21.3-p13.1				
121	Wigner P et al. [160]	2018	281/236	Poland	IDO1	rs10089084	8p11.21				
					KAT1	rs10988134	2q31.1	0,043			
					AADAT	rs1480544	4q33				
					AADAT	rs3824259	4q33				
122	Wigner P et al. [151]	2017	281/229	Poland	NOS2	rs10459953	17q11.2				1.288 (1.012–1.639)
					NOS1	rs1879417	12q24.22				
					NOS2	rs2297518	17q11.2				
					SOD2	rs4880	6q25.3	0,008			
					GPX4	rs713041	19p13.3	< 0.001	6.450 (2.998–13.877)	< 0.001	
					CAT	rs7943316	11p13	0,002	1.757 (1.234–2.499)		1.198 (1.06-1.35)
123	Wigner P et al. [152]	2018	280/230	Poland	TPH1	rs10488682	11p15.1	0,019	1.515 (1.070–2.145)	0,009	
					TPH1	rs1799913	11p15.1	0,042	1.473 (1.013–2.141)	0,04	
					TPH1	rs1800532	11p15.1	0,016	2.416 (1.180–4.947)		1.491(1.179-1.887)
					TPH2	rs4570625	12q21.1	<0.001	5.942 (3.999–8.831)	0,001	
					TPH1	rs623580	11p15.1	0,027	2.092 (1.089–4.021)	< 0.001	
					TPH2	rs7963803	12q21.1	<0.001	2.223 (1.563–3.160)	0,024	0.840 (0.75-0.95)
124	Czarny P et al. [153]	2018	282/303	Poland	SMUG1	rs3087404	12q13.13				
					UNG	rs34259	12q24.11	0,021	1.480 (1.061–2.064)	0,0016	
					DUT	rs4775748	15q21.1				
					MAP2K2	rs10250	19p13.3				
					MAP2K1	rs1174378	15q22.31				
					MAP2K2	rs12459484	19p13.3				

125	Hu Y et al. [154]	2017	425/386	China	MAP2K1	rs1432441	15q22.31	0,025	0.73 (0.60–0.89)		
					MAP2K1	rs1549854	15q22.31	0,005	0.73 (0.60–0.89)		
					MAP2K2	rs350903	19p13.3				
					MAP2K2	rs350913	19p13.3				
					MAP2K1	rs4255740	15q22.31				
					MAP2K1	rs7182853	15q22.31	0,039	0.81 (0.66–0.99)		
					MAP2K2	rs7258366	19p13.3				
126	Mihailova S et al. [170]	2016	80/52	Bulgaria	IL10	1800871	1q32.1				
					TGFB1	rs1800471	19q13.2				
					TNF	rs1800629	6p21.33				
					IL6	rs1800795	7p15.3				
					IL10	rs1800893	1q32.1				1,249 (1.013988-1.539571)
					IL10	rs1800896	1q32.1				1,302 (1.056501-1.604563)
					TGFB1	rs1982073	19q13.2		2,62		
					IFNG	rs2430561	12q15				
127	Pereira PA et al. [34]	2013	190/77	Brazil	AKT1	rs10149779	14q32.33				
					AKT1	rs1130214	14q32.33				
					AKT1	rs2494731	14q32.33				
					AKT1	rs2494738	14q32.33				
					AKT1	rs2494746	14q32.33				
					AKT1	rs3730358	14q32.33	0,006	2.036 (1.06–3.90)	0,003	0.485 (0.286–0.833)
					AKT1	rs3803304	14q32.33				
					AKTIP	rs7189819	16q12.2				
					AKTIP	rs9302648	16q12.2				
128	Gałecka E et al. [155]	2015	179/152	Poland	DIO2	rs12885300 (ORFa-Gly3Asp)	14q31.1				
					DIO2	rs225014 (Thr92Ala)	14q31.1	0,03	0.09 (0.01;-0.82)		
129	Czarny P et al. [156]	2015	257/298	Poland	hOGG1	rs1052133	3p25.3				
					MUTYH	rs3219489	1p34.1				
					NEIL1	rs4462560	15q24.2	0,014	2.894 (1.245–6.728)	0,005	
130	Gałecki P et al. [72]	2013	268/200	Poland	VEGFA	rs2010963 (405G/C)	6p21.1	0,013	1.82 (1.01–3.33)	0,004	
					VEGFA	rs3025039(936 C/T)	6p21.1				
131	Seripa D et al. [157]	2013	218/363	Italy	SLC6A4	rs140701	17q11.2				1.694 (1.228–2.335)
					SLC6A4	rs3813034	17q11.2				
					SLC6A4	rs4795541	17q11.2				
132	Froud A et al. [166]	2017	187/55	Australia	BDNF	rs6265	11p14.1				1.25 (1.10–2.08)
					LIG3	rs1052536	17q12	0,006	1.721 (1.168–2.538)	0,028	
					APEX1	rs1130409	14q11.2				
					PARP1	rs1136410	1q42.12				
					FEN1	rs174538	11q12.2				

133	Czarny P et al. [167]	2016	288/311	Poland	APEX1	rs1760944	14q11.2	0,025	1.445 (1.047–1.994)		
					XRCC1	rs1799782	19q13.31				
					LIG1	rs20579	19q13.33				
					XRCC1	rs25487	19q13.31				
					LIG3	rs4796030	17q12	0,003	2.037 (1.266–3.279)	0,008	
134	Gałecki P et al. [168]	2012	268/200	Poland	KDR	rs1870377 (+1416T/A)	4q12	0,01	2.4 (1.3–4.2)	0,01	
					KDR	rs7667298 (271A/G)	4q12				
135	Taylor WD et al. [161]	2011	54/37	USA	AGTR1	rs5186 (A1166C]	3q24				
					BDNF	rs6265	11p14.1				
136	Wang P et al. [162]	2015	586/586	China	CREB1	rs11904814	2q33.3				
					CREB1	rs2253206	2q33.3				
					CREB1	rs2551941	2q33.3				
					CREB1	rs6740584	2q33.3				
137	He Y et al. [163]	2012	314/252	China	DGCR8	rs3757	22q11.21	0,01		0,03	
					AGO1	rs636832	1p34.3	0,046		0,02	
					GEMIN4	rs7813	17p13.3				
138	Buttenschøn HN et al. [164]	2016	408/289	Denmark	ACE		20p13	0.00057*			
					CRH		17q21.31				
					NR3C1		17q23.3				
					FKBP5		5q31.3				
					CRHR2		6p21.31				
					CRHR1		7p14.3				
					AVP		8q13.1				
					ACE	rs3730025	20p13				0.474 (0.280–0.802)
					ACE	rs4295	20p13				
					ACE	rs4309	20p13	0,0092			
					ACE	rs4311	20p13	0,033			
					ACE	rs4316818	20p13				
					ACE	rs4329	20p13				
					ACE	rs4461142	20p13				
					ACE	rs4646994 (ACE I/D)	20p13	0.046			
					ACE	rs8076157	20p13				
139	Ching-López A et al. [35]	2015	67/500	Spain	CRHR1	rs110402	17q21.31			0,036	
					CRHR1	rs173365	17q21.31			0,027	2.28 (1.26–4.13)
					CRHR1	rs17689966	17q21.31			0,026	
					CRHR1	rs242924	17q21.31			0,033	
					TPH1	rs623580	11p15.1			0,026	
					CRHR1	rs7209436	17q21.31			0,022	
					Between ESD and HTR2A	rs9526236	ESD 13q14.2-HTR2A 13q14.2			0,027	
140	Fan Yuan et al. [165]	2018	568/1034	China	NR3C1	rs10482614	5q31.3				0,614
					MTHFR	rs1801133	1p36.22				
						rs6191					
						rs72557310					

141	Li T et al. [172]	2013	1135/1135	China	FOXP2	rs1358278	7q31.1		
						rs923875	7q31.1		
						rs6466488	7q31.1		0.0195
						rs17137124	7q31.1		
						rs1563408	7q31.1		
						rs2189015	7q31.1		
						rs2396753	7q31.1		
						rs6960610	7q31.1	0.0284	0.0096
						rs10447760	7q31.1	0.000996	0.0128*
						rs9969232	7q31.1		
						rs1456029	7q31.1		
						rs10250103	7q31.1		
