# Supplementary material for: A Systematic Review of Candidate Genes for Major Depression
Source: Medicina (Kaunas). 2022 Feb 14;58(2):285. doi: 10.3390/medicina58020285 (PMC8875554; doi:10.3390/medicina58020285)
Supplement: Supplementary file 1 [file medicina-58-00285-s001.zip › Table S2.pdf]

Table S2. Summary of genotyping and statistical methods used in the eligible studies

No.	Author (Ref)	Year of publication	Participants (N) Case/Control	Genotyping method	Statistics used
1	Yang J et al. [79]	2019	278/287	qPCR	T-test<br>Chi-square test
2	Aldoghachi AF et al. [76]	2019	300/300	HRM	Chi-square test<br>Multivariable Logistic regression<br>Kruskal–Wallis test
3	Chao JK et al. [77]	2018	265/223	PCR (T-ARMS PCR)	Chi-square test<br>Fisher's exact test<br>ANOVA or Independent sample t-test<br>Logistic regression analysis
4	Ren D et al. [37]	2017	568/846	PCR	Chi-square test<br>Logistic regression
5	Liu J et al. [19]	2017	1135/989	qPCR	Chi square test
6	Xie T et al. [80]	2017	437/377	PCR (KASP PCR with a FRET)	Chi square test<br>Logistic regression analysis
7	Bi Y et al. [59]	2017	568/846	Other (MALDI-TOF-MS)	Logistic regression analysis
8	Dadkhah T et al. [38]	2016	250/250	PCR (PCR-RFLP)	Chi-square test<br>Pearson's chi-square test
9	Mahmood S et al. [81]	2016	111/207	PCR (PCR-RFLP)	The Mann Whitney test<br>Chi-square test<br>Logistic regression analysis
10	Pérez-Olmos I et al. [22]	2016	87/131	PCR	The Shapiro-Wilk test<br>Chi- squared test<br>Logistic regression analysis<br>Pearson´s chi- squared test<br>Post hoc power analysis
11	Treutlein J et al. [42]	2017	595/1295	Gene sequencing (NGS)	Exact test<br>Armitage Trend Test
12	Sarubin N et al. [17]	2016	412/634	qPCR	Fisher's exact test
13	Zhang C et al. [82]	2016	772/759	PCR (Multiplex PCR)	Chi-square test or t test<br>ANCOVA
14	Sayadi MA et al. [83]	2016	208/187	PCR	Chi-squared test<br>T test<br>ANOVA<br>Binary logistic regression analysis
15	Sun N et al. [46]	2016	459/412	PCR	Logistic regression analysis
16	Zhang Z et al. [86]	2015	744/767	PCR (Multiplex PCR)	Chi-square test<br>T test<br>ANOVA
17	Zhang L et al. [84]	2015	292/208	PCR (LDR)	Chi-square test for independence<br>Logistic regression analysis
18	Wei Y et al. [169]	2015	456/582	qPCR	Chi-square test
19	Wang Y et al. [60]	2015	388/388	qPCR	Chi square goodness-of-fit test<br>The Pearson chi square analysis or Fisher exact test
20	Liang Y et al. [85]	2015	237/296	PCR (PCR-RFLP)	Chi-square test

21	Du T et al. [23]	2015	738/1098	Other (MALDI-TOF-MS)	Chi-square test
22	Kokut S et al. [89]	2015	144/105	PCR (PCR-RFLP)	Chi-square test<br>ANOVA<br>T test
23	Watanabe S et al. [1]	2015	216/213	PCR	Chi-square test for goodness of fit<br>Fisher's exact test
24	Congiu C et al. [87]	2014	590/441	Gene sequencing (NGS)	Chi-square test<br>Logistic regression analyses<br>ANOVA
25	Zhou Y et al. [88]	2014	313/318	PCR (PCR-RFLP)	The goodness-of-fit chi-square test<br>Pearson's chi-square test<br>Student's t tests<br>ANOVA
26	Hua P et al. [92]	2014	105/485	PCR (PCR-RFLP)	T-test<br>Chi-square test or the Fisher exact test<br>Binary logistic regression analysis
27	McFarquhar M et al. [90]	2014	649/571	Other (MALDI-TOF-MS)	Linear and logistic regression analysis<br>ANOVA
28	Nazree NE et al. [24]	2013	265/332	PCR (PCR-RFLP and ARMS-PCR)	Chi-square test
29	Mocking R et al. [91]	2013	137/73	PCR	Chi-square -tests<br>Linear mixed regression analysis<br>Post-hoc tests
30	Santos M et al. [20]	2014	80/160	qPCR	Chi-square test<br>The Fisher's exact test
31	Wang Y et al. [95]	2013	700/673	PCR (LDR)	Chi-square test<br>Multivariate logistic regression analysis
32	He M et al. [93]	2013	114/224	PCR (PCR-RFLP)	Chi-square analyses<br>Linear or logistic regression analysis<br>ANOVA<br>ANCOVA
33	Evinova A et al. [94]	2012	134/143	PCR (PCR-RFLP)	Chi-square test
34	Cao S et al. [100]	2012	315/278	PCR (LDR)	T-test<br>The Pearson chi-square test
35	Tian W et al. [96]	2012	463/413	PCR (PCR-RFLP) Other (MALDI-TOF MS)	The goodness of-fit chi-square -test<br>The Mann–Whitney U-test<br>Kruskal–Wallis test
36	Minelli A et al. [97]	2012	522/375	Gene sequencing (NGS)	The chi-square test<br>ANOVA
37	Vereczkei A et al. [98]	2019	195/406	qPCR	Chi-square tests<br>Dimensional analysis<br>Linear regression analysis
38	Tao S et al. [99]	2018	185/64	Gene sequencing (NGS)	Logistic regression analysis<br>T test<br>Chi-square test
39	Cao SX et al. [61]	2018	504/303	PCR (LDR)	T-test or ANOVA<br>Chi-square test for goodness
40	Zeng D et al. [101]	2018	225/436	qPCR	T-test<br>Chi-squared test
41	Han KM et al. [55]	2018	95/65	qPCR	Independent t-tests<br>Chi-square tests<br>ANCOVA
42	Wang L et al. [25]	2017	1056/1248	Other (MALDI-TDF-MS)	Chi-square test<br>Chi square test for goodness of fit
43	Wang Q et al. [105]	2017	1045/1235	qPCR	Chi-square test<br>Logistic regression analysis
44	Wang L et al. [106]	2017	67/44	PCR	Student's t test<br>ANOVA<br>Pearson correction analysis
45	Can MŞ et al. [102]	2017	86/89	PCR (PCR-RFLP)	Kolmogorov-Smirnov and Shapiro-Wilk tests<br>Student's t-test or Mann-Whitney U test<br>Chi-square or Fisher's exact tests<br>Pearson or Spearman test

46	Han KM et al. [103]	2017	105/85	qPCR	T-test<br>Chi-square test<br>RM-ANCOVA<br>Pearson's partial correlation
47	Cribb L et al. [104]	2017	154/53	Other (MALDI-TDF-MS)	T-test<br>Chi-square tests<br>Univariate regression analysis<br>ANOVA<br>Logistic regression analysis
48	Wang Y et al. [112]	2017	75/45	qPCR	Chi-square goodness of fit tests<br>ANOVA
49	Xu C et al. [113]	2017	381/291	Other (MALDI-TOF-MS)	Student's t test<br>Pearson's chi-square test<br>The chi-square goodness-of-fit test
50	Han D et al. [114]	2017	274/273	PCR	Chi-square goodness-of-fit test<br>Chi-square test<br>Multiple logistic regression analysis
51	Won E et al. [107]	2017	103/83	qPCR	Chi-square test<br>T-test<br>ANCOVA
52	Tollenaar MS et al. [108]	2017	428/387	Other (Microarray analysis)	ANOVAs<br>Chi square tests<br>Logistic and multiple regression analyses
53	Ma J et al. [109]	2017	512/513	PCR	Chi-square goodness-of-fit test<br>The chi-square test
54	Han KM et al. [110]	2017	114/88	PCR	T-tests<br>Chi-square test<br>ANCOVA<br>Post-hoc analysis<br>The Pearson's partial correlation analysis<br>Hierarchical moderated regression analysis
55	Mandelli L et al. [111]	2017	238/324	Gene sequencing (pyrosequencing)	The Student t test<br>ANOVA<br>Linear or binary logistic regression analyses
56	Bondarenko EA et al. [117]	2016	150/200	Other (MALDI-TOF-MS)<br>qPCR	Chi-square test
57	Ninomiya-Baba M et al. [26]	2016	799/1189	qPCR	Chi-square test for goodness of fit<br>Chi-square test for independence<br>Multiple regression analysis
58	Zhang J et al. [21]	2016	191/2000	qPCR	Pearson's chi-square test<br>Student's t-test<br>Logistic regression
59	Choi S et al. [115]	2016	86/64	PCR	ANCOVA<br>T-tests<br>Chi-square test
60	Mushtaq R et al. [116]	2016	240/160	PCR	Chi-square test<br>Fisher's exact test<br>T-test<br>ANOVA
61	Tatham EL et al. [56]	2016	55/18	PCR (PCR-RFLP)	Fisher exact test<br>T-test<br>Linear regression analysis
62	Wang Y et al. [47]	2016	200/199	PCR	Independent t-test<br>Pearson chi-square test<br>Chi-square test<br>Multivariable binary logistic regression analysis<br>Nonparametric Spearman correlation<br>Stepwise logistic regression analysis
63	Kostic M et al. [48]	2016	77/66	qPCR	ANOVA<br>Post-hoc pairwise comparisons<br>Chi-square test
64	Wen Z et al. [121]	2016	1056/1248	Other (MALDI-TOF-MS)	Chi-square test for goodness of fit<br>Chi-square test for independence<br>Omnibus chi-square test
65	Khan RAW et al. [27]	2016	1056/1248	Other (MALDI-TOF-MS)	Chi-square test
66	Wen Z et al. [122]	2016	1056/1248	Other (MALDI-TOF-MS)	Omnibus chi-squared test

67	Li W et al. [15]	2015	1045/1235	qPCR	Pearson chi-square test<br>Logistic regression test
68	Sublette ME et al. [118]	2016	635/480	Gene sequencing (NGS)	Logistic regression analyses<br>The Cochranx– Mantel – Haenszel test<br>Chi-square test
69	Wei YB et al. [119]	2015	436/1590	PCR	Shapiro-Wilk and the Levene's tests<br>Mann-Whitney U test<br>Kruskal-Wallis test<br>Pearson's chi-square test or the Fisher's exact test<br>ANCOVA
70	Ma J et al. [120]	2015	289/289	qPCR	Chi-square and chi-square goodness-of-fit test
71	He M et al. [126]	2019	334/497	qPCR<br>PCR (LDR)	T-test<br>Chi-square test<br>Pearson's chi-square test<br>Multivariate logistic regression analysis
72	Milaneschi Y et al. [127]	2014	1544/336+2470	Other (Microarray analysis)	Linear and multi-nomial logistic regressions analyses
73	Kitzlerová E et al. [50]	2018	68/90	PCR (PCR-RFLP)	Chi-square test<br>Pearson's chi-square test<br>Logistic regression analysis
74	Won E et al. [123]	2016	52/52	Gene sequencing (NGS)	2-sample t-tests<br>Chi-square test<br>ANCOVA
75	Quteineh L et al. [124]	2016	**CoLaus|PsyCoLaus**<br>rs6510997 - 1431/1916<br>**The radiant study**<br>rs3746266 - 2142/700;<br>rs2075017 - 1352/ 808<br>**NESDA/NTR study**<br>rs3746266 - 1768/2895;<br>rs6510997 - 1768/ 2895	Other (Microarray analysis)<br>Gene sequencing (NGS)<br>Other (Microarray analysis)	Chi-square or Fisher exact tests<br>Multivariable regression analyses<br>Linear regression analysis
76	Ma XC et al. [28]	2013	218/514	Other (MALDI-TOF-MS)	Chi-square analysis<br>Logistic regression analysis
77	Ho PS et al. [49]	2012	40/12	qPCR	Chi-squared test<br>Pearson's correlation coefficients<br>Multiple linear regression analysis
78	Sarmiento-Hernández EI et al. [53]	2019	200/235	PCR	Chi-Square test<br>ANOVA
79	Chang HS et al. [125]	2015	270/204	qPCR	Chi-square test<br>Multiple logistic regression analysis<br>Linear regression analysis<br>Power analysis
80	Lee SM et al. [133]	2018	13/20	PCR	Shapiro-Wilk test<br>Spearman's rank correlation tests<br>Pearson's chi-squared test<br>Independent samples t-test
81	Rao S et al. [51]	2016	36/141	PCR	T test<br>Fisher's exact test<br>Chi-square test<br>Logistic regression analysis
82	Yin H et al. [39]	2016	219/57	Other	Logistic regressions analyses
83	Crisafulli C et al. [29]	2012	145/170	Gene sequencing (pyrosequencing)	Chi-square test<br>ANOVA

84	Gałecka E et al. [71]	2012	181/149	PCR (PCR-RFLP)	Chi square test<br>Post hoc power analysis<br>Logistic regression analysis
85	Elfving B et al. [134]	2012	162/289	Other (MALDI-TOF-MS)	Multiple linear regression analysis<br>Univariate linear regression analyses
86	Chang HS et al. [128]	2015	149/193	qPCR	Chi square test<br>Multiple logistic regression analysis<br>Generalized linear analysis<br>Independent t-test
87	Nielsen MG et al. [129]	2015	414/259	qPCR	Chi-square tests and logistic regression analyses<br>ANOVA<br>The Kruskal–Wallis and<br>Mann–Whitney U non parametric tests
88	Inoue A et al. [130]	2015	280/398	qPCR	Chi-square test<br>ANOVA<br>Dunnett ’s least significant difference test<br>Stepwise regression analysis
89	Traks T et al. [131]	2015	391/389	Gene sequencing	Chi-squared test
90	Li W et al. [132]	2015	1045/1235	qPCR	Pearson chi-square tests<br>Chi-square test
91	Wang Q et al. [76]	2015	1045/1520	qPCR	Chi square tests (goodness fit and omnibus)
92	Hayashi K et al. [171[	2014	30/30	Gene sequencing (NGS)	Chi-square test<br>Unpaired t-test
93	Stacey D et al. [139]	2014	171/512	Other (MALDI-TOF-MS)	The chi square test<br>ANOVA
94	Wang Q et al. [30]	2014	1045/1235	qPCR	Chi-square test<br>Chi-square test for goodness of fit test
95	Frazier TW et al. [140]	2014	48/149	qPCR	Pearson correlation coefficients<br>ANOVA<br>Pearson chi-square test
96	Ozbey G et al. [141]	2014	54/70	PCR (PCR-RFLP)	The two-tailed Pearson's chi-square test and Fisher's exact test
97	Szczepankiewicz A et al. [142]	2014	218/742	qPCR	Two-tailed power analysis
98	Shen X et al. [143]	2014	368/219	PCR (PCR-RFLP)	Chi square test<br>A logistic regression analysis
99	Wang L et al. [18]	2014	517/455	Other (MALDI-TOF-MS)	Chi-square goodness-of-fit test
100	Jia W et al. [135]	2014	409/619	Other (MALDI-TOF-MS)	Chi-square analysis<br>Fisher’s exact test<br>Unconditional logistic regression analysis<br>Kaplan-Meier survival analysis
101	Sasayama D et al. [136]	2013	39/40	PCR	T-test<br>Manne Whitney U test<br>Chi-square for goodness of fit test<br>Chi-square test for independence test<br>Chi-square test<br>Spearman’s rank correlation coefficient
102	Wang X et al. [137]	2013	97/103	PCR (PCR-RFLP)	T-test<br>Chi-square test<br>Spearman’s correlation<br>General linear analysis
103	Zhang C et al. [138]	2013	790/725	PCR	Chi-square test<br>T-test<br>Logistic regression analysis<br>ANCOVA
104	Kalska H et al. [57]	2013	19/19	PCR	Chi square test<br>The independent samples - t test<br>MANCOVA<br>ANCOVA<br>Poisson regression analysis
105	Stacey D et al. [52]	2013	304/312	PCR (PCR-RFLP)	Pearson’s chi-square test

106	Ahdidan J et al. [58]	2013	23/33	PCR	Student t-test<br>F-test<br>Fisher's exact test<br>Pearson's chi square-test<br>Multiple linear regression analysis
107	Cumurcu BE et al. [144]	2013	80/96	qPCR	Pearson chi-square test<br>Fisher exact test
108	Li H et al. [16]	2013	574/642	qPCR	Chi-square test<br>Fisher's exact test
109	Halmai H et al. [145]	2013	195/373	qPCR and PCR (PCR-RFLP)	Chi-square<br>Independent samples t-test<br>Spearman-correlation<br>ANCOVA<br>MANCOVA
110	Mitjans M et al. [146]	2013	319/150	Other (MALDI-TOF-MS)	Chi-square tests<br>Chi-square tests of independence<br>ANOVA
111	Ji W et al. [31]	2012	1135/1135	qPCR	Logistic regression analysis
112	Chen J et al. [147]	2012	488/480	Gene sequencing (NGS)	Goodness-of-fit Chi square test
113	Wang Y et al. [33]	2012	403/475	Gene sequencing (NGS)	Chi square test<br>T test
114	Yang Z et al. [6]	2012	397/473	Gene sequencing (NGS)	Chi square tests<br>Fisher's Exact Test<br>Student's t test<br>ANOVA
115	Firouzabadi N et al. [148]	2012	191/104	PCR	Student's t-test<br>Pearson's chi square<br>Fisher's exact test<br>ANOVA<br>Bonferroni's post-hoc test<br>Logistic regression models
116	Carballedo A et al. [149]	2012	37/42	qPCR	Student's T-test<br>Chi-square test<br>Mann–Whitney U-test<br>ANCOVA
117	Green EK et al. [150]	2012	1159/2592	Other (MALDI-TOF-MS)	Single-locus association test (Cochran-Armitage Trend test)<br>Logistic regression analysis
118	Bobińska K et al. [158]	2016	203/99	qPCR	Chi-square test<br>Mann-Whitney Rank Sum test<br>Unconditional multiple logistic regression analysis
119	Gałecka E et al. [159]	2016	139/69	qPCR	Chi square test<br>Post hoc power analysis<br>Logistic regression analysis<br>Spearman's rank correlation<br>ANOVA
120	Buttenschøn HN et al. [69]	2015	25/46	Other (MALDI-TOF-MS)	Univariate linear regression analysis<br>Wilcoxon two-sample rank-sum test<br>Chi square test
121	Wigner P et al. [160]	2018	281/236	qPCR	The unconditional multiple logistic regression analysis<br>The Shapiro-Wilks test<br>The Mann-Whitney test<br>Student's t test
122	Wigner P et al. [151]	2017	281/229	qPCR	Unconditional multiple logistic regression analysis<br>The Shapiro-Wilk test<br>The Mann-Whitney test or<br>Student's t test
123	Wigner P et al. [152]	2018	280/230	qPCR	Unconditional multiple logistic regression analysis<br>The Shapiro-Wilk test<br>The Mann-Whitney test or<br>Student's t test

124	Czarny P et al. [153]	2018	282/303	qPCR	Chi square test<br>Unconditional multiple logistic regression analysis<br>The bootstrap-boosted multiple logistic regression analysis<br>The cross-validated logistic regression analysis<br>Hosmer-Lemeshow test<br>Shapiro-Wilk test<br>Brown-Forsythe test<br>Student's t test or<br>Mann-Whitney U test
125	Hu Y et al. [154]	2017	425/386	PCR	Chi-square goodness-of-fit test<br>ANOVA
126	Mihailova S et al. [170]	2016	80/52	PCR (PCR-SSP)	Mann–Whitney U test<br>Chi square test<br>Fischer's exact test
127	Pereira PA et al. [34]	2013	190/77	qPCR	Chi-square test
128	Gałecka E et al. [155]	2015	179/152	PCR	Chi square test<br>Post-hoc power analysis<br>Logistic regression model
129	Czarny P et al. [156]	2015	257/298	qPCR	Chi-square test<br>Unconditional multiple logistic regression model
130	Gałecki P et al. [72]	2013	268/200	PCR (PCR–RFLP)	Chi-square test<br>Post-hoc power analysis<br>Logistic regression mode<br>Mann–Whitney U-test or Kruskal–Wallis test
131	Seripa D et al. [157]	2013	218/363	PCR	Fisher's exact test<br>Binary logistic regression analysis<br>Wild chi-square test
132	Froud A et al. [166]	2017	187/55	Other (MALDI-TOF-MS)	Chi-square test<br>ANOVA
133	Czarny P et al. [167]	2016	288/311	qPCR	Chi-square test<br>Logistic regression analysis
134	Gałecki P et al. [168]	2012	268/200	PCR (PCR–RFLP)	Chi-square test<br>Post-hoc power analysis<br>Logistic regression analysis<br>Mann-Whitney test Kruskal–Wallis test
135	Taylor WD et al. [161]	2011	54/37	PCR	Chi square tests<br>General linear models
136	Wang P et al. [162]	2015	586/586	qPCR	Chi-square test<br>T-test<br>Multi-factor variance analysis<br>Logistic regression analysis
137	He Y et al. [163]	2012	314/252	qPCR	Pearson's chi-square test<br>Wilcoxon's rank-sum test
138	Buttenschøn HN et al. [164]	2016	408/289	OTher (MALDI-TOF-MS)	Linear regression analyses<br>Multiple logistic regression analyses
139	Ching-López A et al. [35]	2015	67/500	qPCR	Student's t test
140	Fan Yuan et al. [165]	2018	568/1034	Other (MALDI-TOF-MS)	Pearson's chi square test
141	Li T et al. [172]	2013	1135/1135	qPCR	The chi-squared test<br>Bonferroni correction
